# Infections Caused by Extended-Spectrum *β*-Lactamase Producing* Escherichia Coli* in Systemic Lupus Erythematosus Patients: Prevalence, Risk Factors, and Predictive Model

**DOI:** 10.1155/2018/8296720

**Published:** 2018-11-18

**Authors:** Yuetian Yu, Hui Shen, Cheng Zhu, Ruru Guo, Yuan Gao, Liangjing Lu

**Affiliations:** ^1^Department of Rheumatology, Ren Ji Hospital, School of Medicine, Shanghai Jiao Tong University, Shanghai 200001, China; ^2^Department of Critical Care Medicine, Ren Ji Hospital, School of Medicine, Shanghai Jiao Tong University, Shanghai 200001, China; ^3^Department of Laboratory Medicine, Shanghai East Hospital, Tongji University School of Medicine, Shanghai 200123, China; ^4^Department of Emergency, Rui Jin Hospital, School of Medicine, Shanghai Jiao Tong University, Shanghai 200005, China

## Abstract

**Objective:**

To investigate the prevalence and risk factors of infections caused by Extended-Spectrum *β*-Lactamase (ESBL) producing* Escherichia coli* (*E. coli*) in systemic lupus erythematosus (SLE) patients and develop a predictive model.

**Methods:**

Three hundred and eighty-four consecutive SLE patients with* E. coli* infection were enrolled in this retrospective case control study from January 2012 to December 2017. Prevalence and antimicrobial susceptibility pattern of ESBL producing* E. coli* were analyzed. Multivariate analysis was performed to determine the risk factors. Sensitivity and specificity were obtained at various point cutoffs and area under the receiver operator characteristic curve (AuROC) was determined to confirm the prediction power of the model.

**Results:**

Of the total 384* E. coli *strains tested, 212 (55.2%) produced ESBL. The majority of these isolates were from urine (44.3%). Carbapenems (>80%) and amikacin (89.6%) had good activity against ESBL producing* E. coli*. Eleven variables were identified as independent risk factors for ESBL producing* E. coli *infection including Enterobacteriaceae colonization or infection in preceding year (OR=8.15, 95%CI 5.12–21.71), daily prednisone dose > 30mg (OR=5.48, 95%CI 3.12–13.72), low C3 levels (OR=2.17, 95%CI 1.62–6.71), nosocomial acquired infection (OR=4.12, 95%CI 1.98–8.85), etc. The model developed to predict ESBL producing* E. coli* infection was effective, with the AuROC of 0.840 (95% CI 0.799-0.876).

**Conclusions:**

The prevalence of ESBL producing* E. coli* was increasing with high antibiotics resistance in patients with SLE. The model revealed excellent predictive performance and exhibited a good discrimination.

## 1. Background

Infection is one of the leading causes of mortality and morbidity in patients with systemic lupus erythematosus (SLE). Almost one-third of patients presented infections during follow-up, and more than 40% of deaths were associated with infections in the first five years [1]. Bacterial infections are not uncommon in patients with SLE due to disease activity, high doses of glucocorticoid, and immunosuppressive agents treatment. If the pathogenic bacteria are drug resistance [such as Extended-Spectrum *β*-Lactamase (ESBL) producing* Escherichia coli (E. coli)*], the mortality can be as high as 50% [2].

Despite the tremendous progress in the area of infectious diseases management in SLE patients, the mortality has not decreased substantially in the last two decades [3]. There remained a great deal of questions unanswered especially in ESBL infections. A key component in the management of ESBL infections is the prediction of its occurrence. A predictive model with high accuracy may help to prevent or reduce the risk of ESBL infections in high risk patients. Thus, the present study aimed to determine the prevalence and risk factors of ESBL producing* E. coli* infections in SLE patients and to develop a reliable predictive model.

## 2. Materials and Methods

### 2.1. Research Briefs

This retrospective case control study was performed in the department of rheumatology in Ren Ji hospital which had a total of 90 hospital beds in this department including 16 intensive care unit (ICU) beds. The annual volume of hospitalization in this department was two thousand and three hundred and the annual volume of the outpatients was two hundred thousand.

### 2.2. Study Population

SLE patients were eligible for enrolment if they were diagnosed with infectious diseases caused by* E. coli* from January 2012 to December 2017. Patients were excluded if they have one or more other pathogens detected during their hospital stay. Patients that fulfilled at least 4 criteria of American College of Rheumatology were diagnosed SLE [4]. The infectious diseases include skin and soft tissue infection, pneumonia, bacteremia, and urinary tract infection which were defined in accordance with uniform diagnostic criteria of European Society of Clinical Microbiology and Infectious Diseases (ESCMID) [5]. Nosocomial acquired infection was defined as the infectious diseases acquired after 48h of hospitalization [6]. The pathogen was acquired either from the hospital or community and patients were categorized into ESBL producing group or non-ESBL producing group. The control group of SLE patients without any observed bacterial infection was matched to the* E. coli *infection cases in a 1:1 ratio based on age and gender.

### 2.3. Data Collection and Clinical Assessment

Information of the patients was obtained from the hospital electronic medical records while the antimicrobial susceptibility results were obtained from the hospital microbiological database. The demographics and clinical characteristics of each patient included were age, gender, and infection type. Daily prednisone dose, systemic lupus erythematosus disease activity index (SLEDAI) score, SLE activity, and the level of complement 3 (C3) at the time of infection were recorded. Low C3 level was defined as the concentration was lower than 0.80g/L. Positive anti-dsDNA was defined as the binding ratio was higher than 20%. Antibiotics treatment history, catheters implantation, and previous or ongoing ICU admissions were also documented.

### 2.4. Strains Identification and ESBL Detection

Strains were identified using bioM'erieux Vitek-2 automated system. Antimicrobial susceptibility testing was performed and the breakpoint (susceptible, intermediate, or resistant) was determined according to Enterobacteriaceae M100-S27 provided by the Clinical and Laboratory Standards Institute (CLSI) standards (http://ncipd.org/control/images/NCIPD_docs/CLSI M100-S27.pdf).

ESBL detection was performed by the double disk diffusion using both cefotaxime and ceftazidime alone and in combination with clavulanic acid. An increase in zone size of more than or equal to 5 mm for cefotaxime and ceftazidime with and without clavulanic acid was taken as an indication of ESBL production [7]. Only the first isolated* E. coli *strain from SLE patients was tested in our study.

### 2.5. Statistical Analysis

Statistical analysis was performed using SPSS version 21.0 (IBM for Windows). Data were initially assessed for normality and log-transformed as appropriate. Data between the ESBL producing or not were compared using Chi-square test for equal proportion or Fisher exact test where numbers were small with results presented as percentages (n). Normally distributed variables were compared using Student's t-test and were expressed as means (standard deviations), whereas nonnormally distributed data was compared using Wilcoxon rank-sum test and reported as medians (interquartile range). Risk factors associated with ESBL producing* E. coli* infection were identified by multivariate logistic regression and summarized with odds ratios (ORs) and 95% confidence intervals (95%CIs). These risk factors were incorporated into the predictive model and the performance of the model was displayed as the area under curve (AUC) of the receiver operating characteristic curve (ROC).

Analysis was performed on an intention-to-treat basis and a two sided* p*<0.05 was considered to be statistically significant. Figures were drawn using GraphPad Prism version 6.0 and Medical calculator version 15.0.

## 3. Results

### 3.1. Prevalence and Antibiotics Susceptibility of ESBL Producing E. coli

**Figure 1:**
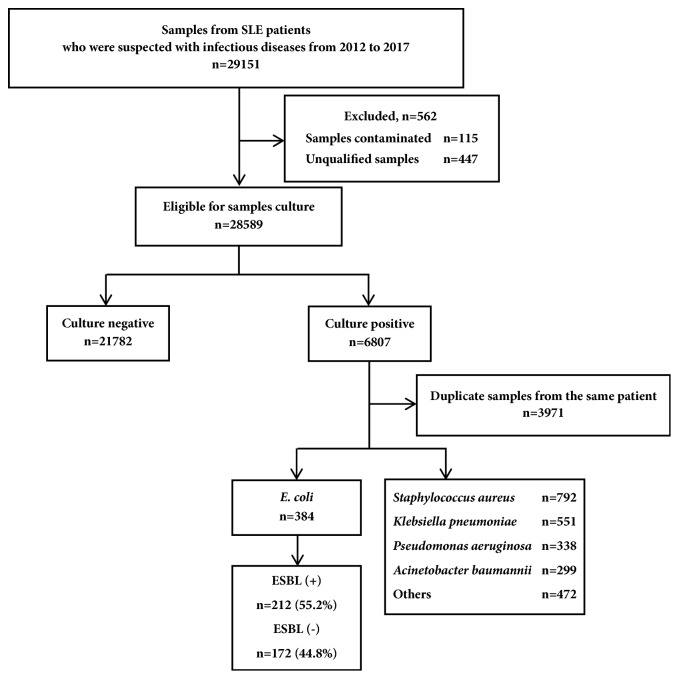
Distribution of the samples from the SLE patients who were suspected infectious diseases. ESBL, Extended-Spectrum *β*-Lactamase; SLE, systemic lupus erythematosus.

**Figure 2:**
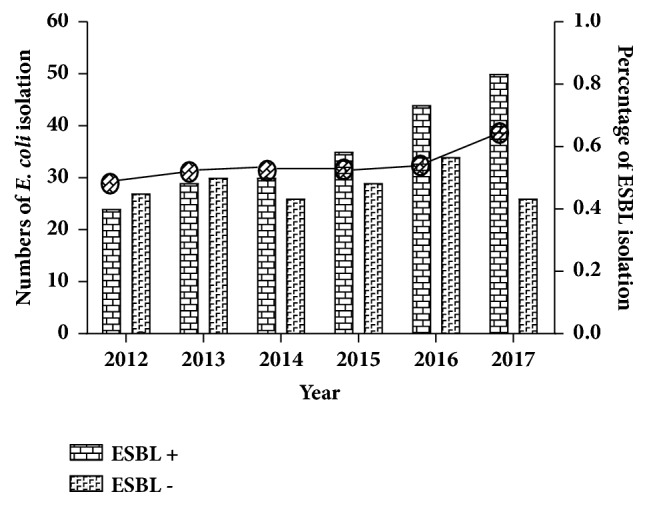
The prevalence of ESBL producing* E. coli* during the six years. Column indicated the number of* E. coli* isolation. Curve indicated the percentage of ESBL isolation. ESBL, Extended-Spectrum *β*-Lactamase.

Totally 29,151 samples from SLE patients who were suspected with infectious diseases from 2012 to 2017 were tested in our study. Six thousand eight hundred and seven samples were shown to have positive culture results and 384* E. coli *strains without duplicate samples were confirmed at last (Figure 1). Of the total 384 isolates of* E. coli*, 212 (55.2%) were confirmed as ESBL producing strains during the last six years. The isolation rates continued to rise from 47.1% in 2012 to 65.8% in 2017 (Figure 2). The distribution of 212 ESBL producing* E. coli* was revealed from the following: 94 in urine, 42 in sputum, 24 in blood, and 52 in other samples (Table 1).

The susceptibility data of* E. coli* were summarized in Table 2. More than 80% of ESBL producing* E. coli* were susceptible to carbapenems as well as amikacin (89.6%) and piperacillin-tazobactam (82.1%).

### 3.2. Clinical Features of ESBL Positive and Negative Groups

As hormone is one of the most important factors that contribute to the incidence of SLE [8], female patients predominated our study population (96.1%). The average age was 49.52 years in ESBL producing group while it was 47.91 in non-ESBL producing group. The rate of ESBL producing* E. coli *isolation was significantly higher in ICU than in ward (16.5% versus 6.4%,* p*=0.002). There was a greatly higher percentage of ESBL producing* E. coli *infection patients with the subsequent clinical features: Enterobacteriaceae colonization or infection in preceding year, nosocomial acquired infection, and catheter implantation (*p*<0.05). The mortality of ESBL producing group was twice as high as the other group (12.7% versus 5.2%,* p*=0.012, Table 3(a)).

As for the SLE status, we found that the SLEDAI score and daily prednisone dose at time of infection was significantly higher in ESBL producing group (*p*<0.001). Low C3 levels might be another factor that was different in the two groups (76.9% versus 52.9%,* p*<0.001). There was no difference in the course of SLE and lymphopenia between the two groups (Table 3(b)).

The relationship between antibiotics prescription within 30 days before the patients infected by* E. coli *was listed in Table 3(c). Statistically significant higher exposures of aminoglycosides, quinolones, and third generation cephalosporins were noted in ESBL producing group (*p*<0.05).

Three hundred and eighty-four SLE patients without infectious diseases were matched to the* E. coli* infection cases in a 1:1 ratio as control group. As it demonstrated that a lower percentage of mechanical ventilation (1.8% versus 6.5%), ICU stay (3.1% versus 11.9%), residence of nursing home (2.1% versus 11.5%), and lupus nephritis (5.7% versus 19.3%) were found in the control group (*p*<0.001), a higher daily dose of prednisone and SLEDAI score were recognized in the* E. coli* infection group and more patients in this group received immunosuppressive treatment (18.2% versus 12.8%,* p*=0.036) during their hospital stay. No significant difference was found in the course of SLE and long of hospital stay,* p*>0.05 (listed in the Supplementary Materials (available here)).

### 3.3. Risk Factors of ESBL Producing E. coli Infection

Risk factors were analyzed in the total of 384 patients. All variables were incorporated into the logistic regression model to build a full model, in which thirteen variables were found to be statistically significant. After binary logistic regression analysis, eleven risk factors were remained significant as displayed in Table 4.

Enterobacteriaceae colonization or infection in preceding year (OR=8.15, 95%CI 5.12–21.71) seemed to be the leading risk factor. SLEDAI score >15 (OR=4.05, 95%CI 2.18–9.36) and daily prednisone dose >30mg at the time of infection (OR=5.48, 95%CI 3.12–13.72) were found to be risk factors for the development of ESBL producing infection (*p*<0.05). Low C3 levels (OR=2.17, 95%CI 1.62–6.71) and nosocomial acquired (OR=4.12, 95%CI 1.98–8.85) were still statistically significant (*p*<0.05) after accounting for other factors in the multivariate logistic regression model. Quinolones prescription and hematological activity remained statistically nonsignificant (*p*>0.05).

### 3.4. Risk Factors of E. coli Infection in Patients with SLE

Based on the previous study [1] and the clinical characteristics of* E. coli *infection SLE patients, risk factors of infection were also evaluated in our study. Enterobacteriaceae colonization or infection in preceding year (OR=6.39, 95%CI 3.96–11.72) was also to be the leading risk factor of* E. coli *infection. High SLEDAI score (>10) and daily prednisone dose (>7.5mg) at the time of hospitalization were also revealed to be risk factors for* E. coli* infection (*p*<0.05). Low C3 levels (OR=3.08, 95%CI 1.07–5.72) were still statistically significant (*p*<0.05) after accounting for other factors in the multivariate logistic regression model. However, immunosuppressive treatment (OR=1.79, 95%CI 0.63–6.05,* p*=0.079) was statistically nonsignificant (*p*>0.05) (listed in the Supplementary Materials (available here)).

### 3.5. Predictive Model for ESBL Producing E. coli Infections

Predictive model for ESBL producing* E. coli* infections was developed based on the risk factors described above.  Table 5 manifested the distribution of cumulative risk factors among ESBL producing or non-ESBL producing group. Zero risk factors were found exclusively in the non-ESBL producing group while no patients with risk factors* ⩾*10 were found in the same group.

**Figure 3:**
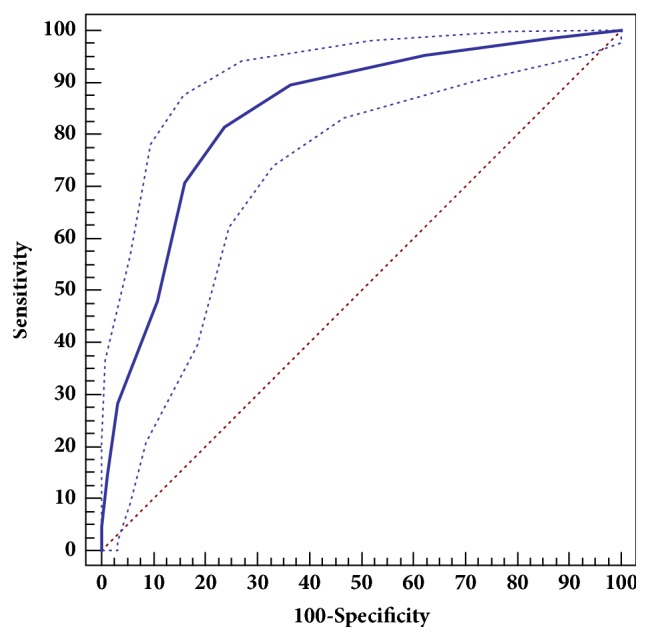
Receiver operating characteristic curves for the model in predicting Extended-Spectrum *β*-Lactamase producing* Escherichia coli* infection.

The AUC of ROC for these data was 0.840 (95%CI 0.799–0.876,* p*<0.001) which indicated that the model displayed excellent predictive power (Figure 3).  Table 6 displayed the predictive efficacy derived from the model. Diagnostic performance parameters were shown for different cutoffs. The predictive model performed best with a cutoff of* ⩾* 4 risk factors.

## 4. Discussion

The prevalence of ESBL varies between countries and institutions as well as underlying diseases. Although some of the studies have address the emergence of ESBL producing enterobacterium in ICU patients [9, 10], there are few researches focusing on patients with SLE. Our study demonstrated the rising trends of ESBL producing* E. coli* in patients with SLE (from 47.1% in 2012 to 65.8% in 2017) which need further concern and more effective methods should be taken to restrain the growing trends.

Multidrug resistance has been reported among ESBL producing organisms and application of antibiotics for these infections is sharply constrained. Our six-year study indicated that the susceptibility of the antibiotics to ESBL producing* E. coli* was far too optimistic. In fact, it seemed that only carbapenems and aminoglycosides appear to be better choices to treat the serious infectious diseases due to* E. coli* with ESBL. Piperacillin-tazobactam (susceptibility was 82.1%) and fosfomycin (susceptibility was 65.1%) might be other alternatives. However, the option was still limited. In 2016, WHO created a priority list of antibiotic resistant bacteria including ESBL producing* E. coli* to support the relative research and development of more effective new drugs [11]. Anyhow, the appropriate prescription of antibiotics still seems to be a corner stone to prevent drug resistance.

It was demonstrated in our study that the rate of ESBL producing strains was much higher in ICU than in wards (16.5% versus 6.4%,* p*=0.002) which conformed to the previous studies [12]. Infection control is a hard job in ICU due to the critical status of the infectious diseases and high antibiotic exposure pressure combined with the immunosuppressive state of the patients. As is well known that prolonged treatment with low concentration of antibiotics will result in multiple antibiotic resistance. So ICUs are identified as the source of drug resistant organisms which can disseminate to the other wards of the hospitals [13]. The mucous membranes of the skin are destroyed by invasive manipulation such as intubation or urethral catheter placement that increase the chance to contact with the ESBL producing strains colonized patients or contaminated objects [14]. We found that almost all the catheters placement were risk factors of acquisition infections caused by* E. coli* with ESBLs including endotracheal tubes (OR=2.19, 95%CI 1.12–5.93) and urethral catheter (OR=2.98, 95%CI 2.01–9.84). Therefore, unnecessary interventional apparatus in ICU should be removed early to prevent hospital acquired infection.

SLE patients with infectious diseases might sometimes be in a critical condition, so the empiric antibiotics coverage should be adequate and appropriate for any possible pathogens. However, indiscriminate antibiotic use has accelerated the incidence of antibiotic resistance in recent years [11]. Nowadays, ESBL producing* E. coli *emerge prominently in SLE patients. Several studies attempted to verify the relationship between antibiotics treatment and acquisition of ESBL producing strains by case control design [15–17]. However, the results of these studies were conflicting due to the difference in study population, sample size, control group selection, etc. In our study, we found the existence of an association between aminoglycosides and third generation cephalosporins usage and the isolation rate of EBSL producing strains in SLE patients.

The disease of SLE itself and its treatment also contribute to infection caused by drug resistant bacteria. Among the treatment regimen, glucocorticoid therapy (both the cumulative dose and the daily dose at the time of infection) is considered to be a major risk factor [18]. As was shown in our study, daily prednisone dose >30mg at the time of infection is an independent risk factor which could contribute to the incidence of ESBL producing pathogens (OR=5.48, 95%CI 3.12–13.72). Immunosuppressive drugs such as cyclophosphamide might disorder the immune system both in humoral and in cellular immunity. Meanwhile, SLE patients also have the feature of defective chemotaxis and phagocytic activity which lead to the alterations during antimicrobial action [19]. To our disappointment, we could not find the difference of the immunosuppressive treatment between the groups although the prescription in the ESBL producing group was relatively high (21.2% versus 14.5%,* p*=0.058). Subgroup analysis of the immunosuppressor is needed in our further study. Furthermore, activity of SLE (with high SLEDAI score) also promotes the infectious complications duo to complement consumption or deficiencies [20]. SLEDAI score >15 means that SLE is in the severely active condition and more glucocorticoid should be prescribed to control the disease. Therefore, it was proved as one of the main risk factors to the incidence of ESBL producing strains (OR=4.05, 95%CI 2.18–9.36).

Some of risk factors were found in our study; some of them were in line with those reported for general population, such as ICU stay during hospitalization, Enterobacteriaceae colonization or infection in preceding year, nosocomial acquired infection, etc. Others factors are specifically associated with the SLE characteristics and its treatment which not only multiply the chances of infectious complications, but also increase the incidence of resistant microorganisms as well. Our predictive model included eleven predictors of ESBL producing* E. coli *infection. Some factors with high levels of odds ratio might have a better predictive power like Enterobacteriaceae colonization or infection in preceding year (OR=8.15) and daily prednisone dose >30mg at the time of infection (OR=5.48), etc.

However, the incidence of ESBL producing* E. coli* infection was a result that many kinds of factors affected together. Thus, if we are intended to screen the SLE patients to determine the possibility of infection caused by ESBL producing* E. coli*, a cutoff point with high sensitivity and low specificity should be adopted.

In our predictive model, the cutoff value was based on the assessment of accuracy, PPV, NPV, sensitivity, and specificity. The best cutoff value for predicting ESBL producing* E. coli *infection was* ⩾*4 points which had an accuracy of 79% and the AUC of ROC for these data was 0.840 (95%CI 0.799–0.876,* p*<0.001).

To the best of our knowledge, it was the first study that described the specific risk factors for ESBL producing* E. coli* in SLE patients and established a predictive model. However, it still had some limitations. The retrospective study was performed in a single center and the number of patients enrolled was relatively low which limited the establishment of subgroups. A major limitation of the study is that the predictive model was not validated in external dataset. The problem of overfitting cannot be fully excluded based on current data. Furthermore, the calibration of the model was not assessed. For some inappropriately specified models, although they reported a good discrimination, the predicted versus observed probability of the event of interest can be poorly aligned in some risk groups [21]. Further multicenter prospective studies are needed to validate our findings and evaluate whether the predictive model can be applied to other immunocompromised populations.

In conclusion, as the rate of ESBL producing* E. coli* isolation was still on the rise, a fast and accurate clinical predictive model for recognition it may improve the empiric antibiotics prescription and decrease the rate of treatment failure as well as the adverse effects. The predictive model can improve the effectiveness of clinical care by applying early targeting of interventions for the SLE patients who is at the risk of ESBL producing* E. coli *infection. Therefore, it could be applied in clinical practice as a tool to prevent drug resistant bacteria infection by helping to identify the high risk patients.

## Figures and Tables

**Table 1:** Samples distribution of ESBL producing and non-ESBL producing strains, n(%).

Specimen type	Total	ESBL (+)	ESBL (-)	*P* value
	n=384	n=212	n=172	
Sputum	76	42 (19.8)	34 (19.8)	0.994
ETA	20	14 (6.6)	6 (3.5)	0.522
BALF	34	20 (9.4)	14 (8.1)	0.754
Urine	174	94 (44.3)	80 (46.5)	0.038
Wound secretion	20	10 (4.7)	10 (5.8)	0.989
Pus	12	4 (3.8)	4 (2.3)	0.876
Blood	48	24 (11.3)	24 (14)	0.583

BALF, bronchoalveolar lavage fluid; ETA, endotracheal aspirate; ESBL, Extended-Spectrum *β*-Lactamase.

**Table 2:** The antimicrobial susceptibility patterns of ESBL producing and nonproducing strains to various antimicrobials, n(%).

Antibiotics	Total	ESBL (+)	ESBL (-)	*P* value
	n=384	n=212	n=172	
Ampicillin	46 (11.9)	0 (0)	46 (27.8)	<0.001
Piperacillin	54 (14.1)	0 (0)	54 (31.4)	<0.001
Ampicillin-sulbactam	120 (31.3)	26 (12.3)	94 (54.7)	<0.001
Piperacillin-tazobactam	346 (90.1)	174 (82.1)	172 (100)	<0.001
Ciprofloxacin	110 (28.6)	20 (9.4)	90 (52.3)	<0.001
Levofloxacin	70 (18.2)	0 (0)	70 (40.7)	<0.001
cefuroxime	114 (29.7)	0 (0)	114 (66.3)	<0.001
ceftazidime	228 (59.4)	56 (26.4)	172 (100)	<0.001
cefepime	226 (58.9)	80 (37.7)	146 (84.9)	<0.001
Aztreonam	260 (67.7)	88 (41.5)	172 (100)	<0.001
Amikacin	354 (92.2)	190 (89.6)	164 (95.3)	0.230
Gentamicin	214 (55.7)	100 (47.2)	114 (66.3)	0.008
Fosfomycin	310 (80.7)	138 (65.1)	172 (100)	<0.001
Trimethoprim-sulfamethoxazole	140 (36.5)	58 (27.4)	82 (47.7)	0.004
Ertapenem	344 (89.6)	172 (81.1)	172 (100)	<0.001
Meropenem	378 (98.4)	206 (97.2)	172 (100)	0.254
Imipenem	378 (98.4)	206 (97.2)	172 (100)	0.254

ESBL, Extended-Spectrum *β*-Lactamase.

**Table:** (a) Demographics and clinical characteristics of SLE patients infected by* E. coli*

Characteristics	ESBL (+)	ESBL (-)	*P* value
(n, % / mean±SD)	n=212	n=172	
Age,yrs	49.5±8.2	47.9±9.8	0.098
Female gender	206 (97.2)	163 (94.8)	0.227
ICU stay during hospitalization	35 (16.5)	11 (6.4)	0.002
Long of hospital stay, days	11.3±4.2	10.5±5.6	0.111
Hospitalization ≥ 48 hours in preceding 90 days	40 (18.9)	33 (19.2)	0.937
Enterobacteriaceae colonization or infection in preceding year	37 (17.5)	6 (3.5)	<0.001
Mechanical ventilation ≥ 48 hours	19 (8.9)	6 (3.5)	0.031
Deep vein catheter ≥ 48 hours	29 (13.7)	18 (10.5)	0.339
Urethral catheter≥ 48 hours	27 (12.7)	8 (4.7)	0.006
Residence of nursing home	33 (15.7)	11 (6.4)	0.005
Nosocomial acquired infection	41 (19.3)	18 (10.5)	0.026
Mortality	27 (12.7)	9 (5.2)	0.012

ESBL, Extended-Spectrum *β*-Lactamase; ICU, intensive care unit.

**Table:** (b) SLE status of the patients infected by* E. coli*

Characteristic (n)	ESBL (+)	ESBL (-)	*P* value
(n, % / mean±SD)	n=212	n=172	
SLE activity at the time of infection			
Lupus nephritis	42 (19.8)	32 (18.6)	0.766
Hematological activity	24 (11.3)	9 (5.2)	0.034
Central nervous system activity	7 (3.3)	3 (1.7)	0.341
Course of SLE, month	42.5±11.3	40.2±14.7	0.084
Immunosuppressive treatment	45 (21.2)	25 (14.5)	0.058
Daily prednisone dose at the time of infection, mg	28.4±7.3	10.6±3.2	<0.001
Positive anti-dsDNA	146 (68.9)	112 (65.1)	0.436
Low C3 levels	163 (76.9)	91 (52.9)	<0.001
Lymphopenia,<1000/ml	74 (34.9)	49 (28.5)	0.181
SLEDAI score	11.4±5.3	5.2±2.1	<0.001

ESBL, Extended-Spectrum *β*-Lactamase; C3, Complement 3; SLE, systemic lupus erythematosus; SLEDAI, systemic lupus erythematosus disease activity index.

**Table:** (c) Antibiotics prescription within 30 days before the patients infected by* E. coli*

Prior Antibiotics (n, %)	ESBL (+)	ESBL (-)	*P* value
	n=212	n=172	
Aminoglycosides	81 (38.2)	27 (15.7)	<0.001
Carbapenem	60 (28.3)	38 (22.1)	0.165
Co-trimoxazole	16 (7.5)	21 (12.2)	0.124
Penicillin group	28 (13.2)	19 (11.0)	0.521
Quinolones	67 (31.6)	38 (22.1)	0.038
First-generation cephalosporins	7 (3.3)	4 (2.3)	0.568
Second-generation cephalosporins	11 (5.2)	7 (4.1)	0.606
Third-generation cephalosporins	55 (25.9)	18 (10.5)	<0.001
Forth-generation cephalosporins	26 (12.3)	17 (9.9)	0.462
*β*-Lactam/*β*-lactamase inhibitors	37 (17.5)	39 (22.6)	0.202

ESBL, Extended-Spectrum *β*-Lactamase.

**Table 4:** Multivariate logistic regression analysis of risk factors for ESBL producing *E. coli*.

Variable	Adjusted OR	95%CI	*P *value
ICU stay during hospitalization	4.16	2.08~11.92	<0.001
Enterobacteriaceae colonization or infection in preceding year	8.15	5.12~21.71	<0.001
Mechanical ventilation *⩾* 48 hours	2.19	1.12~5.93	0.032
Urethral catheter*⩾* 48 hours	2.98	2.01~9.84	0.003
Residence of nursing home	1.89	1.03~4.03	0.037
Nosocomial acquired infection	4.12	1.98~8.85	<0.001
Hematological activity	1.71	0.94~4.03	0.085
Daily prednisone dose at the time of infection			
0mg/day	0.87	0.19~1.58	0.682
<7.5mg/day	2.01	0.82~3.91	0.428
7.5~30mg/day	2.98	0.67~7.94	0.253
>30mg/day	5.48	3.12~13.72	0.017
SLEDAI score			
0~4	0.69	0.47~1.19	0.098
5~9	1.07	0.81~2.83	0.064
10~14	2.84	1.07~5.62	0.061
>15	4.05	2.18~9.36	0.028
Low C3 levels	2.17	1.62~6.71	0.016
Anibiotics prescription within 30 days before infection			
Aminoglycosides	3.19	1.37~7.03	0.023
Quinolones	2.26	0.98~5.02	0.607
Third-generation cephalosporins	5.28	2.06~13.93	<0.001

ESBL, Extended-Spectrum *β*-Lactamase; ICU, intensive care unit; C3, Complement 3; SLEDAI, systemic lupus erythematosus disease activity index.

**Table 5:** Distribution of cumulative risk factors for *E. coli* infected patients.

Number of risk factors	Number of patients, n (%)		
	ESBL (+)	ESBL (-)	Total
0	0 (0)	18 (100)	18
1	3 (12.5)	21 (87.5)	24
2	7 (16.3)	36 (83.7)	43
3	12 (23.1)	40 (76.9)	52
4	17 (45.9)	20 (54.1)	37
5	23 (65.7)	12 (34.3)	35
6	41 (83.7)	8 (16.3)	49
7	49 (80.3)	12 (19.7)	61
8	29 (90.6)	3 (9.4)	32
9	21 (91.3)	2 (8.7)	23
10	7 (100)	0 (0)	7
11	3 (100)	0 (0)	3
Total	212 (55.2)	172 (44.8)	384

ESBL, Extended-Spectrum *β*-Lactamase.

**Table 6:** Performance of the models for predicting ESBL producing *E. coli* at different cutoff values.

Score	TP	FP	TN	FN	Se (%)	Sp (%)	PPV (%)	NPV (%)	Acc (%)
*⩾*1	212	154	18	0	100	11	58	100	60
*⩾*2	209	133	39	3	99	23	61	93	65
*⩾*3	202	97	75	10	95	44	68	86	72
*⩾*4	190	57	115	22	90	67	77	84	79
*⩾*5	173	37	153	39	82	79	82	78	85
*⩾*6	150	25	147	62	71	86	86	70	77
*⩾*7	109	17	155	103	51	90	87	60	69
*⩾*8	60	5	167	162	27	97	92	51	59
*⩾*9	31	2	170	181	15	99	94	48	52
*⩾*10	10	0	172	202	5	100	100	46	47
*⩾*11	3	0	172	209	2	100	100	45	46

TP, number of true positives; FP, number of false positives; FN, number of false negatives; TN, number of true negatives; Se, sensitivity; Sp, specificity; PPV, positive predictive value; NPV, negative predictive value; Acc, rate of accuracy of the risk score model.

## Data Availability

The data used to support the findings of this study including the ESBL detection, antimicrobial susceptibility testing, and basic characteristic of the patients were included within the supplementary information file of this research article.

## Abbreviations

AUC: Area under curve
BALF: Bronchoalveolar lavage fluid
C3: Complement 3
CI: Confidence intervals
ETA: Endotracheal aspirate
ESBL: Extended-Spectrum *β*-Lactamase
ESCMID: European Society of Clinical Microbiology and Infectious Diseases
ICU: Intensive care unit
OR: Odds ratio
ROC: Receiver operating characteristic curve
SLE: Systemic lupus erythematosus
SLEDAI: Systemic lupus erythematosus disease activity index

## Supplementary Materials

Supplementary Materials Supplemental Table 1: clinical characteristics of SLE patients with or without* E. coli* infection. Supplemental Table 2: multivariate logistic regression analysis of risk factors for SLE patients infected by* E. coli*. Supplemental Table 3: the distribution of ESBL producing* E. coli*, number of risk factors of the patients, and the antimicrobial susceptibility testing including ampicillin, piperacillin, ampicillin-sulbactam, piperacillin-tazobactam, ciprofloxacin, levofloxacin, and cefuroxime. Supplemental Table 4: the antimicrobial susceptibility testing of ESBL producing* E. coli* including ceftazidime, cefepime, aztreonam, amikacin, gentamicin, fosfomycin, trimethoprim-sulfamethoxazole, ertapenem, meropenem, and imipenem. Supplemental Table 5: the distribution of non-ESBL producing* E. coli*, number of risk factors of the patients, and the antimicrobial susceptibility testing including ampicillin, piperacillin, ampicillin-sulbactam, piperacillin-tazobactam, ciprofloxacin, levofloxacin, and cefuroxime. Supplemental Table 6: the antimicrobial susceptibility testing of non-ESBL producing* E. coli* including ceftazidime, cefepime, aztreonam, amikacin, gentamicin, fosfomycin, trimethoprim-sulfamethoxazole, ertapenem, meropenem, and imipenem.
