# Supplementary material for: Infections Caused by Extended-Spectrum β-Lactamase Producing Escherichia Coli in Systemic Lupus Erythematosus Patients: Prevalence, Risk Factors, and Predictive Model
Source: Biomed Res Int. 2018 Nov 18;2018:8296720. doi: 10.1155/2018/8296720 (PMC6276506; doi:10.1155/2018/8296720)
Supplement: Supplementary Materials — Supplemental Table 1: clinical characteristics of SLE patients with or without E. coli infection. Supplemental Table 2: multivariate logistic regression analysis of risk factors for SLE patients infected by E. coli. Supplemental Table 3: the distribution of ESBL producing E. coli, number of risk factors of the patients, and the antimicrobial susceptibility testing including ampicillin, piperacillin, ampicillin-sulbactam, piperacillin-tazobactam, ciprofloxacin, levofloxacin, and cefuroxime. Supplemental Table 4: the antimicrobial susceptibility testing of ESBL producing E. coli including ceftazidime, cefepime, aztreonam, amikacin, gentamicin, fosfomycin, trimethoprim-sulfamethoxazole, ertapenem, meropenem, and imipenem. Supplemental Table 5: the distribution of non-ESBL producing E. coli, number of risk factors of the patients, and the antimicrobial susceptibility testing including ampicillin, piperacillin, ampicillin-sulbactam, piperacillin-tazobactam, ciprofloxacin, levofloxacin, and cefuroxime. Supplemental Table 6: the antimicrobial susceptibility testing of non-ESBL producing E. coli including ceftazidime, cefepime, aztreonam, amikacin, gentamicin, fosfomycin, trimethoprim-sulfamethoxazole, ertapenem, meropenem, and imipenem. [file 8296720.f1.pdf]

Supplementary Materials

Supplemental table 1: Clinical characteristics of SLE patients with or without *E.coli* infection

Supplemental table 2: Multivariate logistic regression analysis of risk factors for SLE patients infected by *E.coli*

Supplemental table 3: The distribution of ESBL producing *E.coli*, number of risk factors of the patients and the antimicrobial susceptibility testing including: Ampicillin, Piperacillin, Ampicillin-sulbactam, Piperacillin-tazobactam, Ciprofloxacin, Levofloxacin and cefuroxime.

Supplemental table 4: The antimicrobial susceptibility testing of ESBL producing *E.coli* including: Ceftazidime, cefepime, Aztreonam, Amikacin, Gentamicin, Fosfomycin, Trimethoprim-sulfamethoxazole, Ertapenem, Meropenem and Imipenem.

Supplemental table 5: The distribution of non ESBL producing *E.coli*, number of risk factors of the patients and the antimicrobial susceptibility testing including: Ampicillin, Piperacillin, Ampicillin-sulbactam, Piperacillin-tazobactam, Ciprofloxacin, Levofloxacin and cefuroxime.

Supplemental table 6: The antimicrobial susceptibility testing of non ESBL producing *E.coli* including: Ceftazidime, cefepime, Aztreonam, Amikacin, Gentamicin, Fosfomycin, Trimethoprim-sulfamethoxazole, Ertapenem, Meropenem and Imipenem.

Supplementary table1. Clinical characteristics of SLE patients with or without *E.coli* infection

Characteristics (n, % / mean ±SD)	E.coli infection n=384	Control n=384	*P* value
Age,yrs	48.7 ±7.6	48.3 ±7.4	0.460
Female gender	369 (96.1)	369 (96.1)	1.000
ICU stay during hospitalization	46 (11.9)	12 (3.1)	<0.001
Long of hostital stay,days	10.8 ±4.4	10.7 ±4.8	0.764
Hospitalization ⩾ 48 hours in preceding 90 days	73 (19.0)	60 (15.6)	0.215
Enterobacteriaceae colonization or infection in preceding year	43 (11.2)	6 (1.6)	<0.001
Mechanical ventilation ⩾ 48 hours	25 (6.5)	7 (1.8)	0.001
Deep vein catheter ⩾ 48 hours	47 (12.2)	33 (8.6)	0.098
Urethral catheter⩾ 48 hours	35 (9.1)	9 (2.3)	<0.001
Residence of nursing home	44 (11.5)	8 (2.1)	<0.001
Mortality	36 (9.4)	7 (1.8)	<0.001
SLE activity at the time of hospitalization			
Lupus nephritis	74 (19.3)	22 (5.7)	<0.001
Hematological activity	33 (8.6)	18 (4.7)	0.029
Central nervous system activity	10 (2.6)	9 (2.3)	0.816
Course of SLE, month	41.7 ±12.6	41.3 ±12.7	0.451
Immunosuppressive treatment	70 (18.2)	49 (12.8)	0.036
Daily prednisone dose before hospitalization	21.7 ±5.4	10.7 ±3.1	<0.001
Positive anti-dsDNA	258 (67.2)	233 (60.7)	0.06
Low C3 levels	254 (66.1)	162 (42.2)	<0.001
Lymphopenia,<1000/ml	123 (32.0)	109 (28.4)	0.271
SLEDAI score	8.7 ±3.6	4.2 ±1.9	<0.001

ESBL, Extended-Spectrum β-Lactamase; ICU, Intensive care unit; C3,Complement 3; SLE, Systemic lupus erythematosus; SLEDAI, Systemic Lupus Erythematosus Disease Activity Index;

Supplementary table2. Multivariate logistic regression analysis of risk factors for SLE patients infected by *E.coli*

Variable	Adjusted OR	95%CI	*P* value
ICU stay during hospitalization	3.19	1.72～8.63	<0.001
Enterobacteriaceae colonization or infection in preceding year	6.39	3.96～11.72	<0.001
Mechanical ventilation ≥ 48 hours	2.08	1.06～4.38	0.041
Urethral catheter≥ 48 hours	2.49	1.32～6.74	0.032
Residence of nursing home	1.09	0.51～3.38	0.119
Lupus nephritis	3.13	1.88～6.02	0.031
Low C3 levels	3.08	1.07～5.82	0.039
Immunosuppressive treatment	1.79	0.63～6.05	0.079
Daily prednisone dose at the time of hospitalization			
0mg/day	0.66	0.22～1.86	0.439
<7.5mg/day	1.77	0.75～4.03	0.328
7.5～30mg/day	2.56	1.18～7.41	0.038
>30mg/day	4.99	2.18～10.96	0.019
SLEDAI score			
0～4	0.59	0.32～1.08	0.062
5～9	1.21	0.75～3.44	0.072
10～14	3.16	1.15～6.08	0.043
>15	4.19	2.71～8.02	0.019

ESBL, Extended-Spectrum β-Lactamase; ICU, Intensive care unit; C3,Complement 3; SLE, Systemic lupus erythematosus; SLEDAI, Systemic Lupus Erythematosus Disease Activity Index;

Supplemental table 3

Number	ESBL	Gender	Department	Sample distribution	Bacteria	Number of risks	Ampicillin	Piperacillin	Ampicillin-sulbactam	Piperacillin-tazobactam	Ciprofloxacin	Levofloxacin	cefuroxime
1	+	male	immunology	sputum	e.coli	5	R	R	R	R	R	R	R
2	+	female	immunology	sputum	e.coli	7	R	R	S	S	R	R	R
3	+	female	immunology	sputum	e.coli	7	R	R	R	S	R	R	R
4	+	female	immunology	sputum	e.coli	2	R	R	R	S	S	R	R
5	+	female	immunology	sputum	e.coli	6	R	R	S	S	R	R	R
6	+	female	immunology	sputum	e.coli	7	R	R	R	R	R	R	R
7	+	female	immunology	sputum	e.coli	5	R	R	R	S	R	R	R
8	+	female	immunology	sputum	e.coli	8	R	R	R	S	R	R	R
9	+	female	immunology	sputum	e.coli	6	R	R	S	R	R	R	R
10	+	female	immunology	sputum	e.coli	3	R	R	R	S	R	R	R
11	+	female	immunology	sputum	e.coli	7	R	R	R	S	R	R	R
12	+	female	immunology	sputum	e.coli	6	R	R	S	R	R	R	R
13	+	female	immunology	sputum	e.coli	8	R	R	R	S	S	R	R
14	+	female	immunology	sputum	e.coli	5	R	R	R	S	R	R	R
15	+	female	immunology	sputum	e.coli	7	R	R	S	R	R	R	R
16	+	female	immunology	sputum	e.coli	6	R	R	R	S	R	R	R
17	+	female	immunology	sputum	e.coli	2	R	R	R	S	R	R	R
18	+	female	immunology	sputum	e.coli	8	R	R	S	S	R	R	R
19	+	female	immunology	sputum	e.coli	9	R	R	R	R	R	R	R
20	+	female	immunology	sputum	e.coli	6	R	R	R	S	R	R	R
21	+	female	immunology	sputum	e.coli	3	R	R	S	S	R	R	R
22	+	female	immunology	sputum	e.coli	7	R	R	R	R	R	R	R
23	+	female	immunology	sputum	e.coli	9	R	R	R	S	R	R	R
24	+	female	immunology	sputum	e.coli	1	R	R	R	S	R	R	R
25	+	female	immunology	sputum	e.coli	8	R	R	S	S	S	R	R
26	+	female	immunology	sputum	e.coli	6	R	R	R	S	R	R	R
27	+	female	immunology	sputum	e.coli	8	R	R	R	S	R	R	R
28	+	female	immunology	sputum	e.coli	6	R	R	R	R	R	R	R
29	+	female	immunology	sputum	e.coli	5	R	R	R	S	R	R	R
30	+	female	immunology	sputum	e.coli	7	R	R	S	S	R	R	R
31	+	female	immunology	sputum	e.coli	6	R	R	R	S	R	R	R
32	+	male	immunology	sputum	e.coli	8	R	R	R	S	R	R	R
33	+	female	immunology	sputum	e.coli	5	R	R	R	R	R	R	R
34	+	female	immunology	sputum	e.coli	8	R	R	S	S	R	R	R
35	+	female	immunology	sputum	e.coli	6	R	R	R	S	R	R	R
36	+	female	immunology	sputum	e.coli	9	R	R	S	S	S	R	R
37	+	female	immunology	sputum	e.coli	6	R	R	R	S	R	R	R
38	+	female	immunology	sputum	e.coli	8	R	R	R	R	R	R	R
39	+	female	immunology	sputum	e.coli	3	R	R	R	S	R	R	R
40	+	female	immunology	sputum	e.coli	7	R	R	S	S	R	R	R
41	+	female	immunology	sputum	e.coli	9	R	R	R	S	R	R	R
42	+	female	immunology	sputum	e.coli	5	R	R	R	S	R	R	R
43	+	female	immunology	ETA	e.coli	10	R	R	S	R	R	R	R
44	+	female	immunology	ETA	e.coli	6	R	R	R	S	R	R	R
45	+	female	immunology	ETA	e.coli	9	R	R	R	S	R	R	R
46	+	female	immunology	ETA	e.coli	7	R	R	R	R	S	R	R
47	+	female	immunology	ETA	e.coli	5	R	R	S	S	R	R	R
48	+	female	immunology	ETA	e.coli	10	R	R	R	S	R	R	R
49	+	female	immunology	ETA	e.coli	6	R	R	R	R	R	R	R
50	+	female	immunology	ETA	e.coli	9	R	R	R	S	R	R	R
51	+	female	immunology	ETA	e.coli	6	R	R	S	S	R	R	R
52	+	female	immunology	ETA	e.coli	1	R	R	R	S	R	R	R
53	+	female	immunology	ETA	e.coli	9	R	R	R	S	R	R	R
54	+	female	immunology	ETA	e.coli	6	R	R	R	S	R	R	R
55	+	female	immunology	ETA	e.coli	6	R	R	S	S	R	R	R
56	+	male	immunology	ETA	e.coli	10	R	R	R	S	R	R	R
57	+	female	immunology	BALF	e.coli	5	R	R	R	S	R	R	R
58	+	female	immunology	BALF	e.coli	7	R	R	S	R	R	R	R

59	+	female	immunology	BALF	e.coli	7	R	R	R	S	S	R	R
60	+	female	immunology	BALF	e.coli	10	R	R	R	S	R	R	R
61	+	female	immunology	BALF	e.coli	3	R	R	S	S	R	R	R
62	+	female	immunology	BALF	e.coli	7	R	R	R	S	R	R	R
63	+	female	immunology	BALF	e.coli	10	R	R	R	S	R	R	R
64	+	female	immunology	BALF	e.coli	7	R	R	R	S	R	R	R
65	+	female	immunology	BALF	e.coli	5	R	R	S	S	R	R	R
66	+	female	immunology	BALF	e.coli	10	R	R	R	R	R	R	R
67	+	female	immunology	BALF	e.coli	7	R	R	R	S	R	R	R
68	+	female	immunology	BALF	e.coli	2	R	R	R	S	R	R	R
69	+	female	immunology	BALF	e.coli	7	R	R	S	S	R	R	R
70	+	female	immunology	BALF	e.coli	10	R	R	R	S	S	R	R
71	+	female	immunology	BALF	e.coli	5	R	R	R	S	R	R	R
72	+	female	immunology	BALF	e.coli	7	R	R	R	S	R	R	R
73	+	female	immunology	BALF	e.coli	6	R	R	R	S	R	R	R
74	+	female	immunology	BALF	e.coli	4	R	R	R	S	R	R	R
75	+	female	immunology	BALF	e.coli	11	R	R	R	R	R	R	R
76	+	female	immunology	BALF	e.coli	11	R	R	S	S	R	R	R
77	+	female	immunology	Urine	e.coli	5	R	R	R	S	R	R	R
78	+	female	immunology	Urine	e.coli		R	R	R	S	R	R	R
79	+	female	immunology	Urine	e.coli	3	R	R	R	S	R	R	R
80	+	female	immunology	Urine	e.coli	11	R	R	S	S	R	R	R
81	+	female	immunology	Urine	e.coli	4	R	R	R	S	S	R	R
82	+	female	immunology	Urine	e.coli	9	R	R	R	R	R	R	R
83	+	female	immunology	Urine	e.coli	9	R	R	R	S	R	R	R
84	+	female	immunology	Urine	e.coli	4	R	R	S	S	R	R	R
85	+	female	immunology	Urine	e.coli	7	R	R	R	S	R	R	R
86	+	female	immunology	Urine	e.coli	9	R	R	R	S	R	R	R
87	+	female	immunology	Urine	e.coli	6	R	R	R	S	R	R	R
88	+	female	immunology	Urine	e.coli	9	R	R	S	S	R	R	R
89	+	female	immunology	Urine	e.coli	4	R	R	R	R	R	R	R
90	+	female	immunology	Urine	e.coli	9	R	R	R	S	R	R	R
91	+	female	immunology	Urine	e.coli	6	R	R	R	S	R	R	R
92	+	female	immunology	Urine	e.coli	9	R	R	R	S	R	R	R
93	+	female	immunology	Urine	e.coli	3	R	R	S	S	S	R	R
94	+	female	immunology	Urine	e.coli	9	R	R	R	S	R	R	R
95	+	female	immunology	Urine	e.coli	5	R	R	R	S	R	R	R
96	+	female	immunology	Urine	e.coli	9	R	R	R	R	R	R	R
97	+	female	immunology	Urine	e.coli	7	R	R	R	S	R	R	R
98	+	female	immunology	Urine	e.coli	9	R	R	R	S	R	R	R
99	+	female	immunology	Urine	e.coli	1	R	R	S	S	R	R	R
100	+	female	immunology	Urine	e.coli	7	R	R	R	S	R	R	R
101	+	female	immunology	Urine	e.coli	3	R	R	R	S	R	R	R
102	+	female	immunology	Urine	e.coli	7	R	R	R	S	R	R	R
103	+	female	immunology	Urine	e.coli	6	R	R	R	R	R	R	R
104	+	female	immunology	Urine	e.coli	5	R	R	R	S	R	R	R
105	+	female	immunology	Urine	e.coli	8	R	R	R	S	S	R	R
106	+	female	immunology	Urine	e.coli	6	R	R	R	S	R	R	R
107	+	female	immunology	Urine	e.coli	7	R	R	R	R	R	R	R
108	+	female	immunology	Urine	e.coli	3	R	R	R	S	R	R	R
109	+	female	immunology	Urine	e.coli	9	R	R	R	S	R	R	R
110	+	female	immunology	Urine	e.coli	6	R	R	R	S	R	R	R
111	+	female	immunology	Urine	e.coli	8	R	R	R	S	R	R	R
112	+	female	immunology	Urine	e.coli	5	R	R	R	R	R	R	R
113	+	female	immunology	Urine	e.coli	7	R	R	R	S	R	R	R
114	+	female	immunology	Urine	e.coli	9	R	R	R	S	R	R	R
115	+	female	immunology	Urine	e.coli	3	R	R	R	S	R	R	R
116	+	female	immunology	Urine	e.coli	8	R	R	R	S	S	R	R
117	+	female	immunology	Urine	e.coli	5	R	R	R	S	R	R	R
118	+	female	immunology	Urine	e.coli	7	R	R	R	R	R	R	R
119	+	female	immunology	Urine	e.coli	6	R	R	R	S	R	R	R

120	+	male	immunology	Urine	e.coli	9	R	R	R	S	R	R	R
121	+	female	immunology	Urine	e.coli	7	R	R	R	S	R	R	R
122	+	female	immunology	Urine	e.coli	5	R	R	R	S	R	R	R
123	+	female	immunology	Urine	e.coli	8	R	R	R	S	R	R	R
124	+	female	immunology	Urine	e.coli	8	R	R	R	R	R	R	R
125	+	female	immunology	Urine	e.coli	4	R	R	R	S	R	R	R
126	+	female	immunology	Urine	e.coli	9	R	R	R	S	R	R	R
127	+	female	immunology	Urine	e.coli	5	R	R	R	S	R	R	R
128	+	female	immunology	Urine	e.coli	4	R	R	R	S	S	R	R
129	+	female	immunology	Urine	e.coli	8	R	R	R	S	R	R	R
130	+	female	immunology	Urine	e.coli	6	R	R	R	R	R	R	R
131	+	female	immunology	Urine	e.coli	6	R	R	R	S	R	R	R
132	+	female	immunology	Urine	e.coli	7	R	R	R	S	R	R	R
133	+	female	immunology	Urine	e.coli	4	R	R	R	S	R	R	R
134	+	female	immunology	Urine	e.coli	7	R	R	R	S	R	R	R
135	+	female	immunology	Urine	e.coli	6	R	R	R	R	R	R	R
136	+	female	immunology	Urine	e.coli	8	R	R	R	S	R	R	R
137	+	female	immunology	Urine	e.coli	4	R	R	R	S	R	R	R
138	+	female	immunology	Urine	e.coli	7	R	R	R	S	R	R	R
139	+	female	immunology	Urine	e.coli	5	R	R	R	S	S	R	R
140	+	female	immunology	Urine	e.coli	7	R	R	R	S	R	R	R
141	+	female	immunology	Urine	e.coli	8	R	R	R	R	R	R	R
142	+	female	immunology	Urine	e.coli	7	R	R	R	S	R	R	R
143	+	female	immunology	Urine	e.coli	4	R	R	R	S	R	R	R
144	+	female	immunology	Urine	e.coli	6	R	R	R	S	R	R	R
145	+	female	immunology	Urine	e.coli	7	R	R	R	R	R	R	R
146	+	female	immunology	Urine	e.coli	5	R	R	R	S	R	R	R
147	+	female	immunology	Urine	e.coli	8	R	R	R	S	R	R	R
148	+	female	immunology	Urine	e.coli	4	R	R	R	S	S	R	R
149	+	female	immunology	Urine	e.coli	7	R	R	R	S	R	R	R
150	+	female	immunology	Urine	e.coli	6	R	R	R	R	R	R	R
151	+	female	immunology	Urine	e.coli	6	R	R	R	S	R	R	R
152	+	female	immunology	Urine	e.coli	8	R	R	R	S	R	R	R
153	+	female	immunology	Urine	e.coli	2	R	R	R	S	R	R	R
154	+	female	immunology	Urine	e.coli	8	R	R	R	S	R	R	R
155	+	female	immunology	Urine	e.coli	7	R	R	R	R	R	R	R
156	+	female	immunology	Urine	e.coli	8	R	R	R	S	R	R	R
157	+	female	immunology	Urine	e.coli	4	R	R	R	S	R	R	R
158	+	female	immunology	Urine	e.coli	7	R	R	R	S	R	R	R
159	+	female	immunology	Urine	e.coli	6	R	R	R	R	R	R	R
160	+	female	immunology	Urine	e.coli	6	R	R	R	S	S	R	R
161	+	female	immunology	Urine	e.coli	7	R	R	R	S	R	R	R
162	+	female	immunology	Urine	e.coli	7	R	R	R	S	R	R	R
163	+	female	immunology	Urine	e.coli	4	R	R	R	S	R	R	R
164	+	female	immunology	Urine	e.coli	6	R	R	R	R	R	R	R
165	+	female	immunology	Urine	e.coli	7	R	R	R	S	R	R	R
166	+	female	immunology	Urine	e.coli	7	R	R	R	S	R	R	R
167	+	female	immunology	Urine	e.coli	2	R	R	R	S	R	R	R
168	+	female	immunology	Urine	e.coli	8	R	R	R	S	R	R	R
169	+	female	immunology	Urine	e.coli	6	R	R	R	R	R	R	R
170	+	female	immunology	Urine	e.coli	5	R	R	R	S	S	R	R
171	+	female	immunology	Wound secretion	e.coli	7	R	R	R	S	R	R	R
172	+	female	immunology	Wound secretion	e.coli	4	R	R	R	S	R	R	R
173	+	female	immunology	Wound secretion	e.coli	7	R	R	R	S	R	R	R
174	+	female	immunology	Wound secretion	e.coli	6	R	R	R	R	R	R	R
175	+	female	immunology	Wound secretion	e.coli	8	R	R	R	S	R	R	R
176	+	female	immunology	Wound secretion	e.coli	6	R	R	R	S	R	R	R
177	+	female	immunology	Wound secretion	e.coli	8	R	R	R	S	R	R	R
178	+	female	immunology	Wound secretion	e.coli	6	R	R	R	S	R	R	R
179	+	female	immunology	Wound secretion	e.coli	5	R	R	R	R	R	R	R
180	+	female	immunology	Wound secretion	e.coli	7	R	R	R	S	S	R	R

181	+	female	immunology	Pus	e.coli	7	R	R	R	S	R	R	R
182	+	female	immunology	Pus	e.coli	4	R	R	R	S	R	R	R
183	+	female	immunology	Pus	e.coli	7	R	R	R	S	R	R	R
184	+	female	immunology	Pus	e.coli	8	R	R	R	R	R	R	R
185	+	female	immunology	Blood	e.coli	5	R	R	R	S	R	R	R
186	+	female	immunology	Blood	e.coli	7	R	R	R	S	R	R	R
187	+	female	immunology	Blood	e.coli	6	R	R	R	S	R	R	R
188	+	female	immunology	Blood	e.coli	4	R	R	R	R	R	R	R
189	+	female	immunology	Blood	e.coli	8	R	R	R	S	R	R	R
190	+	female	immunology	Blood	e.coli	8	R	R	R	S	R	R	R
191	+	female	immunology	Blood	e.coli	4	R	R	R	S	R	R	R
192	+	female	immunology	Blood	e.coli	8	R	R	R	S	R	R	R
193	+	female	immunology	Blood	e.coli	6	R	R	R	R	S	R	R
194	+	female	immunology	Blood	e.coli	7	R	R	R	S	R	R	R
195	+	female	immunology	Blood	e.coli	9	R	R	R	S	R	R	R
196	+	female	immunology	Blood	e.coli	3	R	R	R	S	R	R	R
197	+	male	immunology	Blood	e.coli	6	R	R	R	S	R	R	R
198	+	female	immunology	Blood	e.coli	6	R	R	R	S	R	R	R
199	+	female	immunology	Blood	e.coli	7	R	R	R	S	R	R	R
200	+	female	immunology	Blood	e.coli	4	R	R	R	S	R	R	R
201	+	female	immunology	Blood	e.coli	8	R	R	R	S	R	R	R
202	+	male	immunology	Blood	e.coli	5	R	R	R	S	R	R	R
203	+	female	immunology	Blood	e.coli	7	R	R	R	S	S	R	R
204	+	female	immunology	Blood	e.coli	7	R	R	R	S	R	R	R
205	+	female	immunology	Blood	e.coli	3	R	R	R	S	R	R	R
206	+	female	immunology	Blood	e.coli	6	R	R	R	S	R	R	R
207	+	female	immunology	Blood	e.coli	7	R	R	R	S	R	R	R
208	+	female	immunology	Blood	e.coli	8	R	R	R	S	R	R	R
209	+	female	immunology	Blood	e.coli	2	R	R	R	S	R	R	R
210	+	female	immunology	Blood	e.coli	7	R	R	R	S	R	R	R
211	+	female	immunology	Blood	e.coli	6	R	R	R	S	S	R	R
212	+	female	immunology	Blood	e.coli	3	R	R	R	S	R	S	S

Supplemental table 4

Number	ESBL	Gender	Department	Sample distribution	Bacteria	ceftazidime	cefepime	Aztreonam	Amikacin	Gentamicin	Fosfomycin	Trimethoprim-sulfamethoxazole	Ertapenem	Meropenem	Imipenem
1	+	male	immunology	sputum	e.coli	S	R	S	S	S	S	S	R	S	S
2	+	female	immunology	sputum	e.coli	R	S	R	S	R	R	R	S	S	S
3	+	female	immunology	sputum	e.coli	R	R	S	R	R	S	S	S	S	S
4	+	female	immunology	sputum	e.coli	S	S	R	S	S	R	S	S	S	S
5	+	female	immunology	sputum	e.coli	R	S	S	S	R	S	R	R	S	S
6	+	female	immunology	sputum	e.coli	R	S	R	S	S	S	S	S	S	S
7	+	female	immunology	sputum	e.coli	S	R	S	S	R	S	R	S	S	S
8	+	female	immunology	sputum	e.coli	R	R	R	S	S	R	S	R	S	S
9	+	female	immunology	sputum	e.coli	R	R	S	R	R	S	R	S	S	S
10	+	female	immunology	sputum	e.coli	R	S	R	S	S	S	S	R	S	S
11	+	female	immunology	sputum	e.coli	S	R	S	S	R	S	R	S	S	S
12	+	female	immunology	sputum	e.coli	R	R	R	S	S	R	S	S	S	S
13	+	female	immunology	sputum	e.coli	R	R	S	S	R	S	R	S	S	S
14	+	female	immunology	sputum	e.coli	R	S	R	S	S	S	S	R	R	R
15	+	female	immunology	sputum	e.coli	S	R	S	S	R	S	R	S	S	S
16	+	female	immunology	sputum	e.coli	R	S	R	S	S	R	R	S	S	S
17	+	female	immunology	sputum	e.coli	R	R	S	S	R	S	R	R	S	S
18	+	female	immunology	sputum	e.coli	S	R	R	S	S	S	R	S	S	S
19	+	female	immunology	sputum	e.coli	R	S	S	R	R	S	S	S	S	S
20	+	female	immunology	sputum	e.coli	R	R	R	S	S	S	R	S	S	S
21	+	female	immunology	sputum	e.coli	R	R	S	S	S	R	R	R	S	S
22	+	female	immunology	sputum	e.coli	S	R	S	S	R	S	S	S	S	S
23	+	female	immunology	sputum	e.coli	R	S	R	S	S	S	R	S	S	S
24	+	female	immunology	sputum	e.coli	S	R	R	S	R	R	S	S	S	S
25	+	female	immunology	sputum	e.coli	R	R	S	S	S	S	R	S	S	S
26	+	female	immunology	sputum	e.coli	R	R	R	R	R	S	S	S	S	S
27	+	female	immunology	sputum	e.coli	R	S	R	S	R	S	R	R	S	S
28	+	female	immunology	sputum	e.coli	S	R	S	S	S	R	S	S	S	S
29	+	female	immunology	sputum	e.coli	R	R	R	S	R	S	R	S	S	S
30	+	female	immunology	sputum	e.coli	R	R	R	S	S	R	R	S	S	S
31	+	female	immunology	sputum	e.coli	R	S	S	S	R	S	S	R	S	S
32	+	male	immunology	sputum	e.coli	S	R	R	R	R	S	R	S	S	S
33	+	female	immunology	sputum	e.coli	R	R	R	S	R	S	R	S	S	S
34	+	female	immunology	sputum	e.coli	R	R	S	S	S	R	S	R	S	S
35	+	female	immunology	sputum	e.coli	S	R	R	S	R	S	R	S	S	S
36	+	female	immunology	sputum	e.coli	R	S	R	S	S	S	R	S	S	S
37	+	female	immunology	sputum	e.coli	S	R	S	S	R	S	S	S	S	S
38	+	female	immunology	sputum	e.coli	R	R	R	R	R	S	R	S	S	S
39	+	female	immunology	sputum	e.coli	R	S	R	S	S	R	R	R	R	R
40	+	female	immunology	sputum	e.coli	S	R	S	S	R	S	S	S	S	S
41	+	female	immunology	sputum	e.coli	R	S	R	S	S	S	R	S	S	S
42	+	female	immunology	sputum	e.coli	S	S	R	S	R	R	R	S	S	S
43	+	female	immunology	ETA	e.coli	R	R	S	S	S	S	S	R	S	S
44	+	female	immunology	ETA	e.coli	S	R	R	S	S	S	R	S	S	S
45	+	female	immunology	ETA	e.coli	R	S	R	R	R	S	R	S	S	S
46	+	female	immunology	ETA	e.coli	R	R	S	S	R	S	S	R	S	S
47	+	female	immunology	ETA	e.coli	S	R	R	S	S	R	R	S	S	S
48	+	female	immunology	ETA	e.coli	R	R	S	S	R	S	S	S	S	S
49	+	female	immunology	ETA	e.coli	R	S	S	S	S	R	R	S	S	S
50	+	female	immunology	ETA	e.coli	R	R	R	R	R	S	R	S	S	S
51	+	female	immunology	ETA	e.coli	S	R	R	S	R	R	S	S	S	S
52	+	female	immunology	ETA	e.coli	R	S	S	S	S	S	R	S	S	S
53	+	female	immunology	ETA	e.coli	R	R	R	S	R	R	R	R	S	S
54	+	female	immunology	ETA	e.coli	S	R	R	S	S	S	S	S	S	S
55	+	female	immunology	ETA	e.coli	R	S	R	S	R	R	R	S	S	S
56	+	male	immunology	ETA	e.coli	R	R	S	S	S	S	R	S	S	S
57	+	female	immunology	BALF	e.coli	R	R	R	R	R	R	S	S	S	S
58	+	female	immunology	BALF	e.coli	S	R	R	S	S	S	R	R	S	S

59	+	female	immunology	BALF	e.coli	R	S	S	S	S	R	S	S	S	S
60	+	female	immunology	BALF	e.coli	R	R	R	S	R	S	R	S	S	S
61	+	female	immunology	BALF	e.coli	R	R	R	S	S	S	R	S	S	S
62	+	female	immunology	BALF	e.coli	S	S	S	S	R	R	S	S	S	S
63	+	female	immunology	BALF	e.coli	R	R	R	S	S	S	R	R	S	S
64	+	female	immunology	BALF	e.coli	S	S	R	R	R	R	R	S	S	S
65	+	female	immunology	BALF	e.coli	R	R	R	S	R	S	S	S	S	S
66	+	female	immunology	BALF	e.coli	R	R	S	S	S	R	R	S	S	S
67	+	female	immunology	BALF	e.coli	S	S	R	S	R	S	S	S	S	S
68	+	female	immunology	BALF	e.coli	R	R	R	S	S	S	R	R	R	R
69	+	female	immunology	BALF	e.coli	R	R	S	S	R	R	R	S	S	S
70	+	female	immunology	BALF	e.coli	R	S	R	S	S	R	S	S	S	S
71	+	female	immunology	BALF	e.coli	S	R	R	S	R	S	R	R	S	S
72	+	female	immunology	BALF	e.coli	R	R	R	R	R	S	S	S	S	S
73	+	female	immunology	BALF	e.coli	S	S	S	S	S	R	R	S	S	S
74	+	female	immunology	BALF	e.coli	R	R	R	S	R	S	R	S	S	S
75	+	female	immunology	BALF	e.coli	R	R	R	S	S	S	S	R	S	S
76	+	female	immunology	BALF	e.coli	S	S	S	S	R	R	R	S	S	S
77	+	female	immunology	Urine	e.coli	R	R	R	S	S	S	R	S	S	S
78	+	female	immunology	Urine	e.coli	R	R	S	S	S	R	R	S	S	S
79	+	female	immunology	Urine	e.coli	R	S	R	R	R	S	S	S	S	S
80	+	female	immunology	Urine	e.coli	R	R	R	S	S	S	R	S	S	S
81	+	female	immunology	Urine	e.coli	R	R	R	S	S	R	S	R	S	S
82	+	female	immunology	Urine	e.coli	S	S	S	S	R	S	R	S	S	S
83	+	female	immunology	Urine	e.coli	R	R	R	S	S	R	R	S	S	S
84	+	female	immunology	Urine	e.coli	R	R	R	S	R	S	S	S	S	S
85	+	female	immunology	Urine	e.coli	R	R	S	S	S	R	R	S	S	S
86	+	female	immunology	Urine	e.coli	S	S	R	S	R	S	R	R	S	S
87	+	female	immunology	Urine	e.coli	R	R	S	S	S	S	S	S	S	S
88	+	female	immunology	Urine	e.coli	R	S	R	R	R	S	R	S	S	S
89	+	female	immunology	Urine	e.coli	R	S	S	S	R	R	R	S	S	S
90	+	female	immunology	Urine	e.coli	R	R	R	S	S	S	S	R	S	S
91	+	female	immunology	Urine	e.coli	R	S	R	S	R	R	R	S	S	S
92	+	female	immunology	Urine	e.coli	R	R	S	S	S	S	S	S	S	S
93	+	female	immunology	Urine	e.coli	R	S	R	S	R	S	R	S	S	S
94	+	female	immunology	Urine	e.coli	R	R	R	S	R	S	S	S	S	S
95	+	female	immunology	Urine	e.coli	R	S	R	S	S	S	R	S	S	S
96	+	female	immunology	Urine	e.coli	S	S	S	S	R	R	S	R	R	R
97	+	female	immunology	Urine	e.coli	R	R	R	S	R	S	R	S	S	S
98	+	female	immunology	Urine	e.coli	R	S	R	S	S	S	R	S	S	S
99	+	female	immunology	Urine	e.coli	R	R	S	S	S	R	R	S	S	S
100	+	female	immunology	Urine	e.coli	R	R	S	R	R	S	S	S	S	S
101	+	female	immunology	Urine	e.coli	R	S	R	S	S	R	R	S	S	S
102	+	female	immunology	Urine	e.coli	R	R	R	S	R	S	R	S	S	S
103	+	female	immunology	Urine	e.coli	R	R	S	S	R	S	R	S	S	S
104	+	female	immunology	Urine	e.coli	R	R	S	S	S	S	R	S	S	S
105	+	female	immunology	Urine	e.coli	R	S	R	S	R	R	R	S	S	S
106	+	female	immunology	Urine	e.coli	S	R	S	S	R	S	S	S	S	S
107	+	female	immunology	Urine	e.coli	R	R	R	S	S	R	R	S	S	S
108	+	female	immunology	Urine	e.coli	R	S	S	S	R	S	R	S	S	S
109	+	female	immunology	Urine	e.coli	R	R	R	R	S	S	R	R	S	S
110	+	female	immunology	Urine	e.coli	R	R	S	R	R	S	S	S	S	S
111	+	female	immunology	Urine	e.coli	R	R	R	S	S	R	R	S	S	S
112	+	female	immunology	Urine	e.coli	R	S	R	S	S	S	R	S	S	S
113	+	female	immunology	Urine	e.coli	R	R	S	S	R	S	S	S	S	S
114	+	female	immunology	Urine	e.coli	R	S	R	S	R	R	R	S	S	S
115	+	female	immunology	Urine	e.coli	S	R	S	S	S	S	R	S	S	S
116	+	female	immunology	Urine	e.coli	R	S	R	S	R	R	R	S	S	S
117	+	female	immunology	Urine	e.coli	R	R	R	R	S	S	S	S	S	S
118	+	female	immunology	Urine	e.coli	R	R	R	S	R	S	R	S	S	S
119	+	female	immunology	Urine	e.coli	R	S	R	S	S	S	R	R	S	S

120	+	male	immunology	Urine	e.coli	S	R	S	S	R	R	R	S	S	S
121	+	female	immunology	Urine	e.coli	R	R	R	S	S	S	S	S	S	S
122	+	female	immunology	Urine	e.coli	R	S	R	S	S	S	R	S	S	S
123	+	female	immunology	Urine	e.coli	R	R	S	R	R	R	R	S	S	S
124	+	female	immunology	Urine	e.coli	S	S	R	S	S	S	S	S	S	S
125	+	female	immunology	Urine	e.coli	R	R	R	S	R	R	R	S	S	S
126	+	female	immunology	Urine	e.coli	R	S	S	S	R	S	R	S	S	S
127	+	female	immunology	Urine	e.coli	R	R	R	S	S	S	S	S	S	S
128	+	female	immunology	Urine	e.coli	S	S	R	S	R	R	R	S	S	S
129	+	female	immunology	Urine	e.coli	R	R	S	S	S	S	R	S	S	S
130	+	female	immunology	Urine	e.coli	R	R	R	S	S	S	R	S	S	S
131	+	female	immunology	Urine	e.coli	R	S	R	R	R	R	S	S	S	S
132	+	female	immunology	Urine	e.coli	S	R	S	S	R	S	S	S	S	S
133	+	female	immunology	Urine	e.coli	R	R	R	S	S	R	R	S	S	S
134	+	female	immunology	Urine	e.coli	R	S	R	S	R	S	R	S	S	S
135	+	female	immunology	Urine	e.coli	R	R	R	S	S	S	R	R	R	R
136	+	female	immunology	Urine	e.coli	S	R	S	S	R	R	R	S	S	S
137	+	female	immunology	Urine	e.coli	R	R	R	R	S	S	S	S	S	S
138	+	female	immunology	Urine	e.coli	R	S	R	S	R	S	R	R	S	S
139	+	female	immunology	Urine	e.coli	R	R	S	S	S	S	R	S	S	S
140	+	female	immunology	Urine	e.coli	S	S	R	S	R	S	R	S	S	S
141	+	female	immunology	Urine	e.coli	R	R	R	S	R	S	S	S	S	S
142	+	female	immunology	Urine	e.coli	R	S	R	S	S	S	R	S	S	S
143	+	female	immunology	Urine	e.coli	S	R	S	S	S	R	R	S	S	S
144	+	female	immunology	Urine	e.coli	S	S	S	S	R	S	S	S	S	S
145	+	female	immunology	Urine	e.coli	R	R	R	S	S	S	R	R	S	S
146	+	female	immunology	Urine	e.coli	R	S	R	R	R	S	R	S	S	S
147	+	female	immunology	Urine	e.coli	R	R	S	S	R	R	S	R	S	S
148	+	female	immunology	Urine	e.coli	S	S	R	S	S	S	R	S	S	S
149	+	female	immunology	Urine	e.coli	R	R	R	S	R	R	S	S	S	S
150	+	female	immunology	Urine	e.coli	S	S	R	S	S	S	R	S	S	S
151	+	female	immunology	Urine	e.coli	R	R	S	S	R	S	R	S	S	S
152	+	female	immunology	Urine	e.coli	R	S	R	S	S	R	S	S	S	S
153	+	female	immunology	Urine	e.coli	R	R	S	S	S	S	R	S	S	S
154	+	female	immunology	Urine	e.coli	S	S	R	S	R	S	S	R	S	S
155	+	female	immunology	Urine	e.coli	R	R	S	R	R	S	R	S	S	S
156	+	female	immunology	Urine	e.coli	S	S	R	S	S	S	R	S	S	S
157	+	female	immunology	Urine	e.coli	R	R	S	S	R	R	R	R	R	R
158	+	female	immunology	Urine	e.coli	R	S	S	S	S	S	R	S	S	S
159	+	female	immunology	Urine	e.coli	R	R	R	S	R	S	R	S	S	S
160	+	female	immunology	Urine	e.coli	S	R	S	S	S	R	S	S	S	S
161	+	female	immunology	Urine	e.coli	R	S	R	S	R	S	R	S	S	S
162	+	female	immunology	Urine	e.coli	R	R	R	S	S	R	R	S	S	S
163	+	female	immunology	Urine	e.coli	R	R	S	S	S	R	R	R	S	S
164	+	female	immunology	Urine	e.coli	S	S	R	S	R	S	R	S	S	S
165	+	female	immunology	Urine	e.coli	R	R	S	S	S	S	R	S	S	S
166	+	female	immunology	Urine	e.coli	R	R	S	S	R	R	R	S	S	S
167	+	female	immunology	Urine	e.coli	R	R	R	S	R	S	S	R	S	S
168	+	female	immunology	Urine	e.coli	R	S	R	S	S	S	R	S	S	S
169	+	female	immunology	Urine	e.coli	R	R	S	S	R	R	R	S	S	S
170	+	female	immunology	Urine	e.coli	S	S	R	S	S	S	R	S	S	S
171	+	female	immunology	Wound secretion	e.coli	R	R	R	S	S	S	R	R	S	S
172	+	female	immunology	Wound secretion	e.coli	R	S	S	S	R	R	R	S	S	S
173	+	female	immunology	Wound secretion	e.coli	S	R	S	S	S	S	R	S	S	S
174	+	female	immunology	Wound secretion	e.coli	R	R	R	S	R	S	S	S	S	S
175	+	female	immunology	Wound secretion	e.coli	R	S	S	S	R	R	R	S	S	S
176	+	female	immunology	Wound secretion	e.coli	R	R	R	S	S	S	R	S	S	S
177	+	female	immunology	Wound secretion	e.coli	R	S	S	S	R	S	R	R	S	S
178	+	female	immunology	Wound secretion	e.coli	R	R	S	S	S	R	R	S	S	S
179	+	female	immunology	Wound secretion	e.coli	S	S	R	S	R	S	R	S	S	S
180	+	female	immunology	Wound secretion	e.coli	R	R	S	S	S	R	R	S	S	S

181	+	female	immunology	Pus	e.coli	S	R	R	S	R	S	R	R	S	S
182	+	female	immunology	Pus	e.coli	R	S	S	S	S	R	R	S	S	S
183	+	female	immunology	Pus	e.coli	R	R	S	S	R	S	R	S	S	S
184	+	female	immunology	Pus	e.coli	R	R	S	S	R	R	R	S	S	S
185	+	female	immunology	Blood	e.coli	S	S	R	S	S	S	R	S	S	S
186	+	female	immunology	Blood	e.coli	R	R	S	S	S	S	R	R	S	S
187	+	female	immunology	Blood	e.coli	R	R	R	S	R	R	R	S	S	S
188	+	female	immunology	Blood	e.coli	R	S	S	S	S	S	R	S	S	S
189	+	female	immunology	Blood	e.coli	S	R	S	S	R	S	R	S	S	S
190	+	female	immunology	Blood	e.coli	R	S	R	S	S	R	R	S	S	S
191	+	female	immunology	Blood	e.coli	R	R	S	S	R	S	R	S	S	S
192	+	female	immunology	Blood	e.coli	R	S	S	S	S	S	R	S	S	S
193	+	female	immunology	Blood	e.coli	S	R	R	S	R	R	R	R	S	S
194	+	female	immunology	Blood	e.coli	R	R	S	S	R	S	R	S	S	S
195	+	female	immunology	Blood	e.coli	S	S	R	S	S	S	R	S	S	S
196	+	female	immunology	Blood	e.coli	R	R	S	S	R	S	R	S	S	S
197	+	male	immunology	Blood	e.coli	S	R	R	S	S	R	R	S	S	S
198	+	female	immunology	Blood	e.coli	R	S	S	S	R	S	R	S	S	S
199	+	female	immunology	Blood	e.coli	R	R	R	S	S	S	R	S	S	S
200	+	female	immunology	Blood	e.coli	R	S	S	S	R	R	R	R	S	S
201	+	female	immunology	Blood	e.coli	R	R	R	S	R	S	R	S	S	S
202	+	male	immunology	Blood	e.coli	S	S	S	S	R	R	R	S	S	S
203	+	female	immunology	Blood	e.coli	R	R	R	S	S	R	R	R	S	S
204	+	female	immunology	Blood	e.coli	R	R	R	S	R	S	R	S	S	S
205	+	female	immunology	Blood	e.coli	R	S	R	S	S	R	R	S	S	S
206	+	female	immunology	Blood	e.coli	R	R	S	S	R	S	R	S	S	S
207	+	female	immunology	Blood	e.coli	S	R	R	S	R	R	R	S	S	S
208	+	female	immunology	Blood	e.coli	R	S	R	S	S	S	R	S	S	S
209	+	female	immunology	Blood	e.coli	R	R	S	S	R	R	R	S	S	S
210	+	female	immunology	Blood	e.coli	R	S	R	S	S	S	R	S	S	S
211	+	female	immunology	Blood	e.coli	S	R	S	S	S	R	R	R	S	S
212	+	female	immunology	Blood	e.coli	R	R	R	S	R	S	R	S	S	S

Supplemental table 5

Number	ESBL	Gender	Department	Sample distribution	Bacteria	Number of risks	Ampicillin	Piperacillin	Ampicillin-sulbactam	Piperacillin-tazobactam	Ciprofloxacin	Levofloxacin	cefuroxime
1	negative	female	immunology	sputum	e.coli	1	S	S	S	S	S	S	S
2	negative	female	immunology	sputum	e.coli	3	R	S	S	S	R	R	S
3	negative	female	immunology	sputum	e.coli	4	R	R	R	S	S	R	S
4	negative	female	immunology	sputum	e.coli	7	R	R	S	S	R	S	R
5	negative	female	immunology	sputum	e.coli	1	S	S	S	S	R	R	S
6	negative	female	immunology	sputum	e.coli	0	R	R	R	S	S	S	S
7	negative	female	immunology	sputum	e.coli	5	R	R	S	S	S	R	S
8	negative	female	immunology	sputum	e.coli	1	S	S	S	S	S	S	R
9	negative	female	immunology	sputum	e.coli	3	R	R	R	S	R	R	S
10	negative	male	immunology	sputum	e.coli	7	S	R	S	S	S	S	S
11	negative	female	immunology	sputum	e.coli	0	R	R	S	S	R	R	R
12	negative	female	immunology	sputum	e.coli	8	R	R	R	S	R	S	S
13	negative	female	immunology	sputum	e.coli	3	S	S	S	S	S	R	R
14	negative	female	immunology	sputum	e.coli	4	R	R	R	S	S	R	S
15	negative	female	immunology	sputum	e.coli	1	R	R	S	S	R	S	S
16	negative	female	immunology	sputum	e.coli	9	S	S	S	S	R	R	S
17	negative	female	immunology	sputum	e.coli	3	R	R	R	S	S	S	R
18	negative	female	immunology	sputum	e.coli	5	R	R	S	S	R	R	S
19	negative	female	immunology	sputum	e.coli	0	R	R	R	S	S	S	S
20	negative	female	immunology	sputum	e.coli	7	S	S	S	S	S	R	S
21	negative	female	immunology	sputum	e.coli	3	R	R	R	S	R	S	R
22	negative	female	immunology	sputum	e.coli	4	R	R	S	S	S	S	S
23	negative	female	immunology	sputum	e.coli	1	R	R	S	S	R	R	S
24	negative	female	immunology	sputum	e.coli	3	S	S	S	S	R	R	S
25	negative	female	immunology	sputum	e.coli	5	R	R	R	S	S	S	R
26	negative	female	immunology	sputum	e.coli	0	R	S	S	S	R	R	S
27	negative	female	immunology	sputum	e.coli	4	R	R	R	S	S	S	S
28	negative	female	immunology	sputum	e.coli	1	S	S	S	S	S	R	R
29	negative	male	immunology	sputum	e.coli	1	R	R	R	S	R	R	S
30	negative	female	immunology	sputum	e.coli	8	R	R	S	S	R	R	S
31	negative	female	immunology	sputum	e.coli	2	R	R	R	S	S	S	R
32	negative	female	immunology	sputum	e.coli	3	S	S	S	S	R	R	S
33	negative	female	immunology	sputum	e.coli	4	R	R	R	S	S	S	S
34	negative	female	immunology	sputum	e.coli	7	R	R	S	S	S	R	R
35	negative	female	immunology	ETA	e.coli	2	R	R	S	S	R	S	S
36	negative	female	immunology	ETA	e.coli	0	R	R	R	S	R	R	S
37	negative	female	immunology	ETA	e.coli	3	S	S	S	S	S	S	R
38	negative	female	immunology	ETA	e.coli	5	R	R	R	S	S	R	S
39	negative	female	immunology	ETA	e.coli	1	R	S	S	S	R	S	S
40	negative	female	immunology	ETA	e.coli	2	S	S	S	S	R	R	R
41	negative	female	immunology	BALF	e.coli	0	R	R	S	S	S	R	S
42	negative	female	immunology	BALF	e.coli	3	R	R	S	S	R	S	S
43	negative	female	immunology	BALF	e.coli	2	R	R	R	S	S	R	R
44	negative	female	immunology	BALF	e.coli	4	R	R	S	S	R	S	S
45	negative	female	immunology	BALF	e.coli	1	R	R	S	S	S	R	S
46	negative	male	immunology	BALF	e.coli	7	R	R	S	S	S	S	R
47	negative	female	immunology	BALF	e.coli	2	R	R	R	S	R	R	S
48	negative	female	immunology	BALF	e.coli	0	S	S	S	S	R	S	S
49	negative	female	immunology	BALF	e.coli	4	R	R	R	S	S	R	R
50	negative	female	immunology	BALF	e.coli	4	R	R	R	S	R	S	S
51	negative	female	immunology	BALF	e.coli	2	R	R	S	S	S	R	S

52	negative	female	immunology	BALF	e.coli	8	R	R	R	S	S	R	R
53	negative	female	immunology	BALF	e.coli	3	S	S	S	S	S	S	S
54	negative	female	immunology	BALF	e.coli	4	R	R	R	S	R	R	S
55	negative	female	immunology	Urine	e.coli	2	R	S	S	S	S	S	R
56	negative	female	immunology	Urine	e.coli	0	R	R	R	S	S	R	S
57	negative	female	immunology	Urine	e.coli	5	S	S	S	S	R	R	R
58	negative	female	immunology	Urine	e.coli	1	R	R	R	S	S	S	S
59	negative	male	immunology	Urine	e.coli	3	R	R	S	S	S	R	S
60	negative	female	immunology	Urine	e.coli	2	R	R	R	S	R	R	R
61	negative	female	immunology	Urine	e.coli	4	S	S	S	S	S	S	S
62	negative	female	immunology	Urine	e.coli	2	R	R	R	S	R	R	S
63	negative	female	immunology	Urine	e.coli	4	R	R	S	S	S	R	R
64	negative	female	immunology	Urine	e.coli	1	S	S	S	S	R	R	S
65	negative	female	immunology	Urine	e.coli	3	R	R	R	S	S	S	S
66	negative	female	immunology	Urine	e.coli	0	S	R	S	S	R	R	R
67	negative	female	immunology	Urine	e.coli	3	R	R	R	S	S	S	S
68	negative	female	immunology	Urine	e.coli	2	R	R	R	S	S	R	S
69	negative	female	immunology	Urine	e.coli	6	R	R	R	S	R	R	R
70	negative	female	immunology	Urine	e.coli	2	S	S	S	S	S	R	S
71	negative	female	immunology	Urine	e.coli	7	R	R	R	S	R	R	R
72	negative	female	immunology	Urine	e.coli	3	R	R	R	S	S	R	S
73	negative	female	immunology	Urine	e.coli	3	S	S	S	S	S	S	S
74	negative	female	immunology	Urine	e.coli	2	R	R	R	S	R	R	S
75	negative	female	immunology	Urine	e.coli	0	R	R	R	S	S	R	R
76	negative	female	immunology	Urine	e.coli	1	S	S	S	S	S	R	S
77	negative	female	immunology	Urine	e.coli	2	R	R	R	S	R	R	S
78	negative	female	immunology	Urine	e.coli	6	R	R	R	S	S	S	S
79	negative	female	immunology	Urine	e.coli	3	S	S	S	S	R	R	R
80	negative	female	immunology	Urine	e.coli	2	R	R	R	S	S	R	S
81	negative	male	immunology	Urine	e.coli	6	R	R	R	S	R	S	S
82	negative	female	immunology	Urine	e.coli	2	R	R	R	S	S	R	R
83	negative	female	immunology	Urine	e.coli	3	S	S	S	S	S	R	S
84	negative	female	immunology	Urine	e.coli	0	R	R	R	S	R	S	S
85	negative	female	immunology	Urine	e.coli	6	R	R	S	S	S	R	R
86	negative	female	immunology	Urine	e.coli	2	S	R	S	S	R	R	S
87	negative	female	immunology	Urine	e.coli	1	R	R	R	S	S	S	S
88	negative	female	immunology	Urine	e.coli	3	R	R	S	S	R	R	S
89	negative	female	immunology	Urine	e.coli	2	R	R	R	S	R	R	R
90	negative	female	immunology	Urine	e.coli	6	S	S	S	S	S	R	S
91	negative	female	immunology	Urine	e.coli	3	R	R	R	S	R	S	S
92	negative	female	immunology	Urine	e.coli	4	R	R	S	S	S	R	S
93	negative	female	immunology	Urine	e.coli	2	S	S	S	S	R	R	R
94	negative	female	immunology	Urine	e.coli	0	R	R	R	S	S	S	S
95	negative	female	immunology	Urine	e.coli	7	R	R	S	S	R	R	S
96	negative	male	immunology	Urine	e.coli	3	S	R	S	S	S	R	S
97	negative	female	immunology	Urine	e.coli	1	R	R	R	S	R	S	S
98	negative	female	immunology	Urine	e.coli	4	R	R	R	S	S	R	R
99	negative	female	immunology	Urine	e.coli	2	S	S	S	S	R	R	S
100	negative	female	immunology	Urine	e.coli	3	R	R	R	S	S	S	S
101	negative	female	immunology	Urine	e.coli	9	R	R	R	S	R	R	S
102	negative	female	immunology	Urine	e.coli	2	S	S	S	S	S	R	R
103	negative	female	immunology	Urine	e.coli	3	R	R	S	S	S	S	S
104	negative	female	immunology	Urine	e.coli	4	R	R	R	S	R	R	S

105	negative	female	immunology	Urine	e.coli	0	S	S	S	S	S	R	R
106	negative	female	immunology	Urine	e.coli	2	R	S	S	S	R	R	S
107	negative	female	immunology	Urine	e.coli	3	R	R	R	S	S	S	R
108	negative	female	immunology	Urine	e.coli	1	R	S	S	S	R	R	S
109	negative	female	immunology	Urine	e.coli	6	S	R	R	S	S	R	R
110	negative	female	immunology	Urine	e.coli	3	R	S	S	S	S	S	S
111	negative	female	immunology	Urine	e.coli	7	R	R	R	S	S	S	S
112	negative	female	immunology	Urine	e.coli	2	R	R	S	S	R	R	R
113	negative	female	immunology	Urine	e.coli	1	S	S	S	S	S	S	S
114	negative	female	immunology	Urine	e.coli	3	R	R	R	S	R	R	S
115	negative	female	immunology	Urine	e.coli	2	R	R	R	S	S	R	S
116	negative	female	immunology	Urine	e.coli	0	S	S	S	S	S	S	R
117	negative	female	immunology	Urine	e.coli	3	R	R	S	S	R	R	S
118	negative	male	immunology	Urine	e.coli	6	R	R	R	S	S	R	S
119	negative	female	immunology	Urine	e.coli	2	R	R	R	S	R	S	S
120	negative	female	immunology	Urine	e.coli	7	S	S	S	S	S	R	R
121	negative	female	immunology	Urine	e.coli	3	R	R	S	S	S	R	S
122	negative	female	immunology	Urine	e.coli	1	R	R	R	S	R	R	S
123	negative	female	immunology	Urine	e.coli	3	R	R	R	S	S	S	S
124	negative	female	immunology	Urine	e.coli	2	S	S	S	S	R	R	R
125	negative	female	immunology	Urine	e.coli	1	R	R	S	S	S	R	S
126	negative	female	immunology	Urine	e.coli	4	R	R	R	S	R	S	S
127	negative	female	immunology	Urine	e.coli	3	R	R	S	S	S	R	R
128	negative	female	immunology	Urine	e.coli	2	S	S	S	S	R	S	R
129	negative	female	immunology	Urine	e.coli	7	R	S	S	S	S	S	S
130	negative	female	immunology	Urine	e.coli	0	R	R	R	S	R	S	R
131	negative	female	immunology	Urine	e.coli	3	R	R	R	S	S	R	S
132	negative	female	immunology	Urine	e.coli	2	S	S	S	S	R	S	S
133	negative	female	immunology	Urine	e.coli	3	R	R	R	S	S	R	R
134	negative	female	immunology	Urine	e.coli	2	R	R	S	S	R	S	S
135	negative	female	immunology	Wound secretion	e.coli	1	R	R	R	S	S	S	R
136	negative	female	immunology	Wound secretion	e.coli	4	S	S	S	S	R	R	R
137	negative	female	immunology	Wound secretion	e.coli	3	R	R	R	S	S	R	S
138	negative	female	immunology	Wound secretion	e.coli	2	R	S	S	S	R	S	S
139	negative	female	immunology	Wound secretion	e.coli	5	R	R	R	S	S	S	R
140	negative	female	immunology	Wound secretion	e.coli	1	S	S	S	S	R	R	S
141	negative	female	immunology	Wound secretion	e.coli	3	R	R	R	S	S	S	R
142	negative	female	immunology	Wound secretion	e.coli	2	R	R	S	S	R	R	S
143	negative	female	immunology	Wound secretion	e.coli	0	R	R	S	S	S	S	R
144	negative	female	immunology	Wound secretion	e.coli	5	S	S	S	S	R	R	S
145	negative	male	immunology	Pus	e.coli	1	R	R	R	S	S	S	R
146	negative	female	immunology	Pus	e.coli	3	R	R	S	S	R	R	S
147	negative	female	immunology	Pus	e.coli	2	R	R	R	S	S	R	R
148	negative	female	immunology	Pus	e.coli	4	S	S	S	S	R	S	S
149	negative	female	immunology	Blood	e.coli	0	R	R	R	S	S	R	R
150	negative	female	immunology	Blood	e.coli	3	R	R	S	S	R	S	S
151	negative	female	immunology	Blood	e.coli	5	S	S	S	S	S	R	S
152	negative	female	immunology	Blood	e.coli	2	R	R	R	S	R	S	R
153	negative	female	immunology	Blood	e.coli	4	R	R	R	S	S	R	S
154	negative	female	immunology	Blood	e.coli	3	S	S	S	S	R	S	R
155	negative	female	immunology	Blood	e.coli	5	R	R	R	S	S	R	S
156	negative	female	immunology	Blood	e.coli	2	R	R	S	S	R	S	S
157	negative	female	immunology	Blood	e.coli	7	S	S	S	S	R	R	R

158	negative	female	immunology	Blood	e.coli	5	R	R	R	S	S	S	S
159	negative	female	immunology	Blood	e.coli	2	R	S	S	S	R	R	S
160	negative	female	immunology	Blood	e.coli	4	R	R	R	S	S	S	R
161	negative	female	immunology	Blood	e.coli	3	S	S	S	S	R	R	S
162	negative	female	immunology	Blood	e.coli	5	R	R	R	S	R	S	S
163	negative	female	immunology	Blood	e.coli	0	R	R	S	S	S	R	S
164	negative	female	immunology	Blood	e.coli	7	R	S	S	S	R	S	R
165	negative	female	immunology	Blood	e.coli	3	R	R	R	S	R	R	S
166	negative	female	immunology	Blood	e.coli	2	S	S	S	S	R	S	S
167	negative	female	immunology	Blood	e.coli	4	R	R	R	S	R	R	R
168	negative	male	immunology	Blood	e.coli	3	R	R	R	S	S	R	S
169	negative	female	immunology	Blood	e.coli	6	R	S	S	S	R	R	R
170	negative	female	immunology	Blood	e.coli	3	R	R	S	S	R	S	S
171	negative	female	immunology	Blood	e.coli	5	R	R	R	S	R	R	S
172	negative	female	immunology	Blood	e.coli	2	R	S	S	S	S	S	R

Supplemental table 6

Number	ESBL	Gender	Department	Sample distribution	Bacteria	ceftazidime	cefepime	Aztreonam	Amikacin	Gentamicin	Fosfomycin	Trimethoprim-sulfamethoxazole	Ertapenem	Meropenem	Imipenem
1	negative	female	immunology	sputum	e.coli	S	R	S	S	R	S	S	S	S	S
2	negative	female	immunology	sputum	e.coli	S	S	S	S	S	S	R	S	S	S
3	negative	female	immunology	sputum	e.coli	S	S	S	S	S	S	S	S	S	S
4	negative	female	immunology	sputum	e.coli	S	S	S	S	S	S	R	S	S	S
5	negative	female	immunology	sputum	e.coli	S	S	S	S	R	S	S	S	S	S
6	negative	female	immunology	sputum	e.coli	S	S	S	S	S	S	S	S	S	S
7	negative	female	immunology	sputum	e.coli	S	S	S	S	R	S	R	S	S	S
8	negative	female	immunology	sputum	e.coli	S	S	S	R	R	S	S	S	S	S
9	negative	female	immunology	sputum	e.coli	S	R	S	S	S	S	R	S	S	S
10	negative	male	immunology	sputum	e.coli	S	S	S	S	S	S	S	S	S	S
11	negative	female	immunology	sputum	e.coli	S	S	S	S	S	S	S	S	S	S
12	negative	female	immunology	sputum	e.coli	S	S	S	S	R	S	R	S	S	S
13	negative	female	immunology	sputum	e.coli	S	S	S	S	S	S	S	S	S	S
14	negative	female	immunology	sputum	e.coli	S	S	S	S	S	S	R	S	S	S
15	negative	female	immunology	sputum	e.coli	S	S	S	S	S	S	S	S	S	S
16	negative	female	immunology	sputum	e.coli	S	R	S	R	R	S	S	S	S	S
17	negative	female	immunology	sputum	e.coli	S	S	S	S	S	S	R	S	S	S
18	negative	female	immunology	sputum	e.coli	S	S	S	S	R	S	S	S	S	S
19	negative	female	immunology	sputum	e.coli	S	S	S	S	S	S	S	S	S	S
20	negative	female	immunology	sputum	e.coli	S	S	S	S	S	S	R	S	S	S
21	negative	female	immunology	sputum	e.coli	S	S	S	S	R	S	S	S	S	S
22	negative	female	immunology	sputum	e.coli	S	S	S	S	S	S	S	S	S	S
23	negative	female	immunology	sputum	e.coli	S	R	S	S	S	S	S	S	S	S
24	negative	female	immunology	sputum	e.coli	S	S	S	S	S	S	R	S	S	S
25	negative	female	immunology	sputum	e.coli	S	S	S	R	R	S	S	S	S	S
26	negative	female	immunology	sputum	e.coli	S	S	S	S	S	S	R	S	S	S
27	negative	female	immunology	sputum	e.coli	S	S	S	S	R	S	S	S	S	S
28	negative	female	immunology	sputum	e.coli	S	S	S	S	S	S	S	S	S	S
29	negative	male	immunology	sputum	e.coli	S	R	S	S	R	S	R	S	S	S
30	negative	female	immunology	sputum	e.coli	S	S	S	S	S	S	S	S	S	S
31	negative	female	immunology	sputum	e.coli	S	S	S	S	R	S	R	S	S	S
32	negative	female	immunology	sputum	e.coli	S	S	S	S	S	S	S	S	S	S
33	negative	female	immunology	sputum	e.coli	S	S	S	S	R	S	S	S	S	S
34	negative	female	immunology	sputum	e.coli	S	S	S	S	S	S	R	S	S	S
35	negative	female	immunology	ETA	e.coli	S	R	S	S	S	S	S	S	S	S
36	negative	female	immunology	ETA	e.coli	S	S	S	S	S	S	R	S	S	S
37	negative	female	immunology	ETA	e.coli	S	S	S	S	S	S	S	S	S	S
38	negative	female	immunology	ETA	e.coli	S	S	S	S	R	S	R	S	S	S
39	negative	female	immunology	ETA	e.coli	S	S	S	S	S	S	S	S	S	S
40	negative	female	immunology	ETA	e.coli	S	S	S	S	S	S	R	S	S	S
41	negative	female	immunology	BALF	e.coli	S	R	S	S	R	S	S	S	S	S
42	negative	female	immunology	BALF	e.coli	S	S	S	S	S	S	S	S	S	S
43	negative	female	immunology	BALF	e.coli	S	S	S	R	R	S	R	S	S	S
44	negative	female	immunology	BALF	e.coli	S	S	S	S	S	S	R	S	S	S
45	negative	female	immunology	BALF	e.coli	S	S	S	S	S	S	S	S	S	S
46	negative	male	immunology	BALF	e.coli	S	S	S	S	R	S	R	S	S	S
47	negative	female	immunology	BALF	e.coli	S	R	S	S	S	S	S	S	S	S
48	negative	female	immunology	BALF	e.coli	S	S	S	S	S	S	S	S	S	S
49	negative	female	immunology	BALF	e.coli	S	S	S	S	R	S	R	S	S	S
50	negative	female	immunology	BALF	e.coli	S	S	S	S	S	S	R	S	S	S
51	negative	female	immunology	BALF	e.coli	S	S	S	S	R	S	S	S	S	S

52	negative	female	immunology	BALF	e.coli	S	S	S	S	S	S	R	S	S	S
53	negative	female	immunology	BALF	e.coli	S	S	S	S	S	S	R	S	S	S
54	negative	female	immunology	BALF	e.coli	S	R	S	S	S	S	S	S	S	S
55	negative	female	immunology	Urine	e.coli	S	S	S	S	S	S	R	S	S	S
56	negative	female	immunology	Urine	e.coli	S	S	S	S	R	S	S	S	S	S
57	negative	female	immunology	Urine	e.coli	S	S	S	S	S	S	S	S	S	S
58	negative	female	immunology	Urine	e.coli	S	S	S	S	S	S	R	S	S	S
59	negative	male	immunology	Urine	e.coli	S	S	S	S	R	S	S	S	S	S
60	negative	female	immunology	Urine	e.coli	S	R	S	S	S	S	R	S	S	S
61	negative	female	immunology	Urine	e.coli	S	S	S	S	R	S	R	S	S	S
62	negative	female	immunology	Urine	e.coli	S	S	S	S	S	S	S	S	S	S
63	negative	female	immunology	Urine	e.coli	S	S	S	R	R	S	S	S	S	S
64	negative	female	immunology	Urine	e.coli	S	S	S	S	S	S	R	S	S	S
65	negative	female	immunology	Urine	e.coli	S	S	S	S	S	S	S	S	S	S
66	negative	female	immunology	Urine	e.coli	S	R	S	S	S	S	R	S	S	S
67	negative	female	immunology	Urine	e.coli	S	S	S	S	R	S	S	S	S	S
68	negative	female	immunology	Urine	e.coli	S	S	S	S	S	S	R	S	S	S
69	negative	female	immunology	Urine	e.coli	S	S	S	S	R	S	R	S	S	S
70	negative	female	immunology	Urine	e.coli	S	S	S	S	S	S	R	S	S	S
71	negative	female	immunology	Urine	e.coli	S	S	S	S	R	S	S	S	S	S
72	negative	female	immunology	Urine	e.coli	S	S	S	S	S	S	R	S	S	S
73	negative	female	immunology	Urine	e.coli	S	S	S	S	S	S	S	S	S	S
74	negative	female	immunology	Urine	e.coli	S	R	S	S	R	S	S	S	S	S
75	negative	female	immunology	Urine	e.coli	S	S	S	S	S	S	R	S	S	S
76	negative	female	immunology	Urine	e.coli	S	S	S	S	R	S	R	S	S	S
77	negative	female	immunology	Urine	e.coli	S	S	S	S	S	S	S	S	S	S
78	negative	female	immunology	Urine	e.coli	S	S	S	S	R	S	R	S	S	S
79	negative	female	immunology	Urine	e.coli	S	S	S	S	S	S	R	S	S	S
80	negative	female	immunology	Urine	e.coli	S	S	S	S	R	S	S	S	S	S
81	negative	male	immunology	Urine	e.coli	S	S	S	S	S	S	R	S	S	S
82	negative	female	immunology	Urine	e.coli	S	R	S	S	S	S	R	S	S	S
83	negative	female	immunology	Urine	e.coli	S	S	S	S	R	S	S	S	S	S
84	negative	female	immunology	Urine	e.coli	S	S	S	S	S	S	R	S	S	S
85	negative	female	immunology	Urine	e.coli	S	S	S	S	S	S	S	S	S	S
86	negative	female	immunology	Urine	e.coli	S	S	S	R	R	S	R	S	S	S
87	negative	female	immunology	Urine	e.coli	S	S	S	S	S	S	R	S	S	S
88	negative	female	immunology	Urine	e.coli	S	S	S	S	S	S	S	S	S	S
89	negative	female	immunology	Urine	e.coli	S	S	S	S	R	S	R	S	S	S
90	negative	female	immunology	Urine	e.coli	S	R	S	S	S	S	S	S	S	S
91	negative	female	immunology	Urine	e.coli	S	S	S	S	S	S	R	S	S	S
92	negative	female	immunology	Urine	e.coli	S	S	S	S	R	S	S	S	S	S
93	negative	female	immunology	Urine	e.coli	S	S	S	S	S	S	R	S	S	S
94	negative	female	immunology	Urine	e.coli	S	S	S	S	S	S	R	S	S	S
95	negative	female	immunology	Urine	e.coli	S	S	S	S	R	S	S	S	S	S
96	negative	male	immunology	Urine	e.coli	S	S	S	S	S	S	R	S	S	S
97	negative	female	immunology	Urine	e.coli	S	R	S	S	S	S	R	S	S	S
98	negative	female	immunology	Urine	e.coli	S	S	S	S	R	S	S	S	S	S
99	negative	female	immunology	Urine	e.coli	S	S	S	S	S	S	R	S	S	S
100	negative	female	immunology	Urine	e.coli	S	S	S	R	R	S	R	S	S	S
101	negative	female	immunology	Urine	e.coli	S	S	S	S	S	S	S	S	S	S
102	negative	female	immunology	Urine	e.coli	S	S	S	S	R	S	R	S	S	S
103	negative	female	immunology	Urine	e.coli	S	R	S	S	S	S	S	S	S	S
104	negative	female	immunology	Urine	e.coli	S	S	S	S	R	S	R	S	S	S

105	negative	female	immunology	Urine	e.coli	S	S	S	S	S	S	R	S	S	S
106	negative	female	immunology	Urine	e.coli	S	S	S	S	S	S	S	S	S	S
107	negative	female	immunology	Urine	e.coli	S	S	S	S	S	S	R	S	S	S
108	negative	female	immunology	Urine	e.coli	S	S	S	S	R	S	R	S	S	S
109	negative	female	immunology	Urine	e.coli	S	R	S	S	S	S	S	S	S	S
110	negative	female	immunology	Urine	e.coli	S	S	S	S	R	S	R	S	S	S
111	negative	female	immunology	Urine	e.coli	S	S	S	S	S	S	S	S	S	S
112	negative	female	immunology	Urine	e.coli	S	S	S	S	R	S	R	S	S	S
113	negative	female	immunology	Urine	e.coli	S	S	S	S	S	S	S	S	S	S
114	negative	female	immunology	Urine	e.coli	S	S	S	S	S	S	R	S	S	S
115	negative	female	immunology	Urine	e.coli	S	R	S	S	R	S	S	S	S	S
116	negative	female	immunology	Urine	e.coli	S	S	S	S	S	S	R	S	S	S
117	negative	female	immunology	Urine	e.coli	S	S	S	S	S	S	S	S	S	S
118	negative	male	immunology	Urine	e.coli	S	S	S	S	S	S	S	S	S	S
119	negative	female	immunology	Urine	e.coli	S	S	S	R	R	S	S	S	S	S
120	negative	female	immunology	Urine	e.coli	S	S	S	S	S	S	R	S	S	S
121	negative	female	immunology	Urine	e.coli	S	R	S	S	R	S	S	S	S	S
122	negative	female	immunology	Urine	e.coli	S	S	S	S	S	S	R	S	S	S
123	negative	female	immunology	Urine	e.coli	S	S	S	S	S	S	S	S	S	S
124	negative	female	immunology	Urine	e.coli	S	S	S	S	R	S	R	S	S	S
125	negative	female	immunology	Urine	e.coli	S	S	S	S	S	S	S	S	S	S
126	negative	female	immunology	Urine	e.coli	S	R	S	S	S	S	R	S	S	S
127	negative	female	immunology	Urine	e.coli	S	S	S	S	S	S	S	S	S	S
128	negative	female	immunology	Urine	e.coli	S	S	S	S	R	S	R	S	S	S
129	negative	female	immunology	Urine	e.coli	S	S	S	S	S	S	R	S	S	S
130	negative	female	immunology	Urine	e.coli	S	S	S	S	S	S	S	S	S	S
131	negative	female	immunology	Urine	e.coli	S	R	S	S	R	S	R	S	S	S
132	negative	female	immunology	Urine	e.coli	S	S	S	S	S	S	S	S	S	S
133	negative	female	immunology	Urine	e.coli	S	S	S	S	S	S	R	S	S	S
134	negative	female	immunology	Urine	e.coli	S	S	S	S	S	S	S	S	S	S
135	negative	female	immunology	Wound secretion	e.coli	S	S	S	S	R	S	R	S	S	S
136	negative	female	immunology	Wound secretion	e.coli	S	S	S	S	S	S	S	S	S	S
137	negative	female	immunology	Wound secretion	e.coli	S	R	S	S	S	S	R	S	S	S
138	negative	female	immunology	Wound secretion	e.coli	S	S	S	S	S	S	S	S	S	S
139	negative	female	immunology	Wound secretion	e.coli	S	S	S	S	S	S	R	S	S	S
140	negative	female	immunology	Wound secretion	e.coli	S	S	S	S	S	S	S	S	S	S
141	negative	female	immunology	Wound secretion	e.coli	S	R	S	S	S	S	R	S	S	S
142	negative	female	immunology	Wound secretion	e.coli	S	S	S	S	R	S	S	S	S	S
143	negative	female	immunology	Wound secretion	e.coli	S	S	S	S	S	S	S	S	S	S
144	negative	female	immunology	Wound secretion	e.coli	S	S	S	S	S	S	R	S	S	S
145	negative	male	immunology	Pus	e.coli	S	S	S	S	S	S	S	S	S	S
146	negative	female	immunology	Pus	e.coli	S	S	S	S	R	S	R	S	S	S
147	negative	female	immunology	Pus	e.coli	S	R	S	S	S	S	S	S	S	S
148	negative	female	immunology	Pus	e.coli	S	S	S	S	S	S	R	S	S	S
149	negative	female	immunology	Blood	e.coli	S	S	S	S	S	S	R	S	S	S
150	negative	female	immunology	Blood	e.coli	S	S	S	S	S	S	S	S	S	S
151	negative	female	immunology	Blood	e.coli	S	S	S	S	R	S	R	S	S	S
152	negative	female	immunology	Blood	e.coli	S	S	S	S	S	S	R	S	S	S
153	negative	female	immunology	Blood	e.coli	S	S	S	S	S	S	R	S	S	S
154	negative	female	immunology	Blood	e.coli	S	R	S	S	S	S	S	S	S	S
155	negative	female	immunology	Blood	e.coli	S	S	S	S	R	S	R	S	S	S
156	negative	female	immunology	Blood	e.coli	S	S	S	S	S	S	R	S	S	S
157	negative	female	immunology	Blood	e.coli	S	S	S	S	S	S	R	S	S	S

158	negative	female	immunology	Blood	e.coli	S	S	S	S	S	S	S	S	S	S
159	negative	female	immunology	Blood	e.coli	S	S	S	S	R	S	R	S	S	S
160	negative	female	immunology	Blood	e.coli	S	S	S	S	S	S	R	S	S	S
161	negative	female	immunology	Blood	e.coli	S	S	S	S	S	S	R	S	S	S
162	negative	female	immunology	Blood	e.coli	S	S	S	S	R	S	S	S	S	S
163	negative	female	immunology	Blood	e.coli	S	S	S	S	S	S	R	S	S	S
164	negative	female	immunology	Blood	e.coli	S	S	S	S	S	S	R	S	S	S
165	negative	female	immunology	Blood	e.coli	S	S	S	S	R	S	S	S	S	S
166	negative	female	immunology	Blood	e.coli	S	S	S	S	S	S	S	S	S	S
167	negative	female	immunology	Blood	e.coli	S	R	S	S	S	S	R	S	S	S
168	negative	male	immunology	Blood	e.coli	S	S	S	S	R	S	R	S	S	S
169	negative	female	immunology	Blood	e.coli	S	S	S	S	S	S	S	S	S	S
170	negative	female	immunology	Blood	e.coli	S	S	S	S	S	S	R	S	S	S
171	negative	female	immunology	Blood	e.coli	S	S	S	S	S	S	R	S	S	S
172	negative	female	immunology	Blood	e.coli	S	S	S	S	R	S	S	S	S	S
